# Neutrophil-mediated clinical nanodrug for treatment of residual tumor after focused ultrasound ablation

**DOI:** 10.1186/s12951-021-01087-w

**Published:** 2021-10-29

**Authors:** Jian Shen, Junnian Hao, Yini Chen, Hairong Liu, Jianrong Wu, Bing Hu, Yan Wang, Yuanyi Zheng, Xiaojun Cai

**Affiliations:** 1 Department of Ultrasound in Medicine, Shanghai Institute of Ultrasound in Medicine, Shanghai Jiao Tong University Affiliated Sixth People’s Hospital, 600 Yishan Road, 200233 Shanghai, China; 2 State Key Laboratory of Oncogenes and Related Genes, Shanghai Jiao Tong University School of Medicine, 200032 Shanghai, China

## Abstract

**Background:**

The risk of local recurrence after high-intensity focused ultrasound (HIFU) is relatively high, resulting in poor prognosis of malignant tumors. The combination of HIFU with traditional chemotherapy continues to have an unsatisfactory outcome because of off-site drug uptake.

**Results:**

Herein, we propose a strategy of inflammation-tendency neutrophil-mediated clinical nanodrug targeted therapy for residual tumors after HIFU ablation. We selected neutrophils as carriers and PEGylated liposome doxorubicin (PLD) as a model chemotherapeutic nanodrug to form an innovative cell therapy drug (PLD@NEs). The produced PLD@NEs had a loading capacity of approximately 5 µg of PLD per 10^6^ cells and maintained the natural characteristics of neutrophils. The targeting performance and therapeutic potential of PLD@NEs were evaluated using Hepa1-6 cells and a corresponding tumor-bearing mouse model. After HIFU ablation, PLD@NEs were recruited to the tumor site by inflammation (most in 4 h) and released PLD with inflammatory stimuli, leading to targeted and localized postoperative chemotherapy.

**Conclusions:**

This effective integrated method fully leverages the advantages of HIFU, chemotherapy and neutrophils to attract more focus on the practice of improving existing clinical therapies.

**Graphical Abstract:**

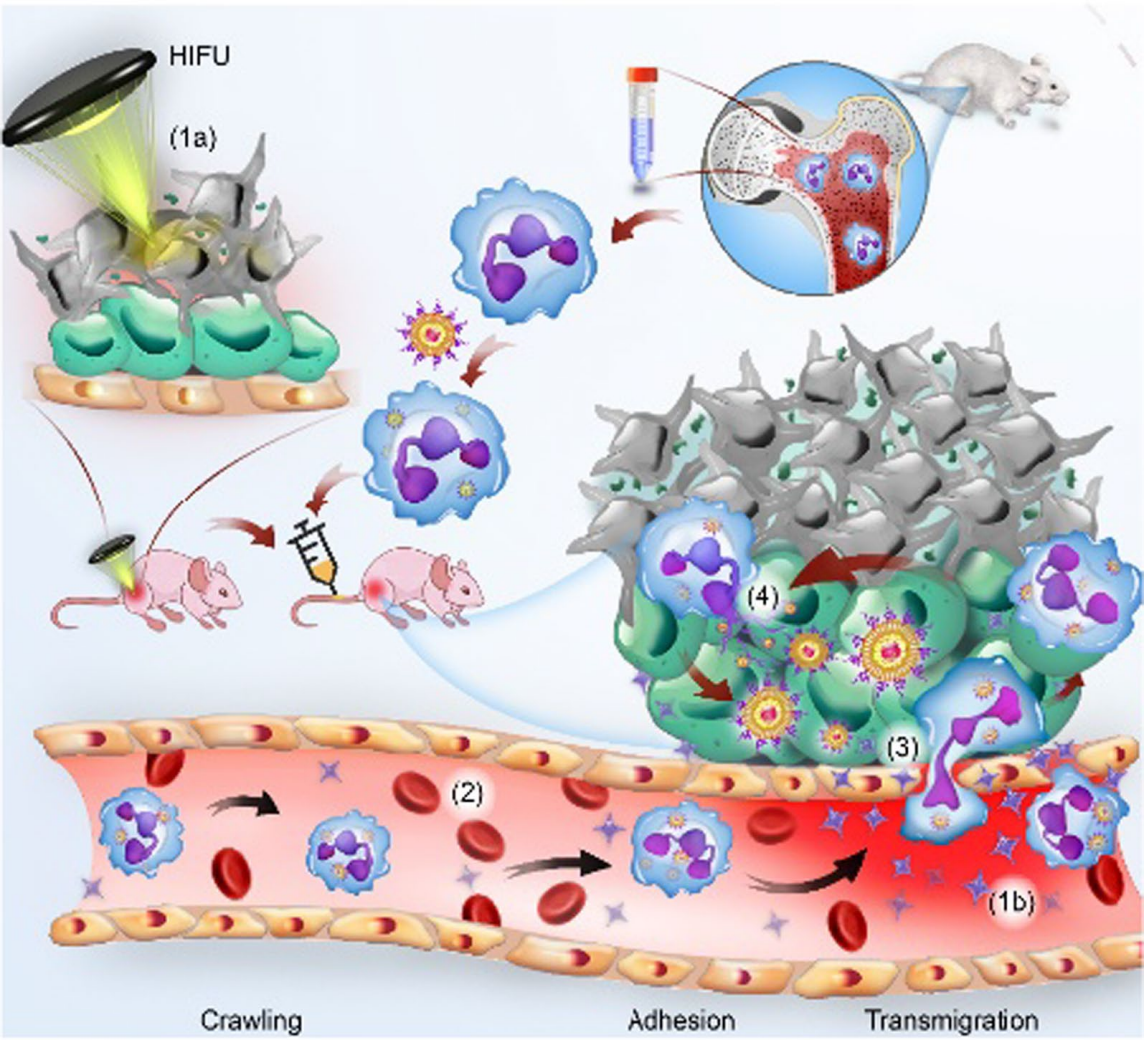

**Supplementary Information:**

The online version contains supplementary material available at 10.1186/s12951-021-01087-w.

## Background

Radiofrequency ablation (RFA), microwave ablation (MWA) and cryoablation are the most commonly used ablation techniques for the clinical treatment of solid tumors. High-intensity focused ultrasound (HIFU) is the only noninvasive hyperthermic ablation modality [1]. Thermal damage and mechanical destruction of tissue are the main principal mechanisms responsible for HIFU-induced tissue necrosis. In the process of ablation, multiple ultrasound beams are focused on a target area to produce a temperature of up to 60 ℃, directly leading to coagulation necrosis of the ablated tissue [2]. HIFU has been routinely used in the clinical treatment of prostate disease and gynecological tumors [2–4]. Notably, HIFU is also being explored as a promising hyperthermic technology for potential application in the clinical treatment of solid tumors such as thyroid tumors, liver cancer, and kidney cancer [5–8]. However, as for other ablation techniques, incomplete ablation is still the main limitation and challenge of HIFU. Incomplete ablation can be caused by several factors: (a) poor detection of the tumor borders using current imaging techniques, especially for small tumors; (b) loss of accurate detection caused by the respiratory motion of patients; (c) scattering or absorption of acoustic waves at the gas or bone interface in the sound field; and (d) a “heat-sink” effect, also known as the “cool-down” effect, that occurs when heat in the treatment region is absorbed by flowing blood, thus decreasing HIFU efficacy [1, 9]. The survival of residual tumor cells after incomplete ablation will eventually lead to tumor recurrence. Therefore, eliminating residual tumor cells to suppress tumor recurrence is of great importance for improving therapeutic outcomes. In addition, an ablation-associated inflammatory response can be observed after ablation with HIFU, as indicated by infiltration of immune cells around the ablated lesion [10]. It has been reported that the presentation of immune cells such as macrophages can enhance systemic antitumor immunity [11]. However, it has also been reported that infiltrating macrophages in the tumor environment can polarize to M2-phenotype tumor-associated macrophages, contributing to tumor progression and even tumor metastasis [12]. Although inflammation after ablation is a double-edged sword, it provided us with a new idea for drug delivery: targeting the tumor’s inflammatory microenvironment after HIFU ablation for drug delivery and using the postoperative response to improve the integrity of HIFU treatment.

Previous studies have provided insights into inflammation-mediated drug delivery based on neutrophils loaded with nanodrugs for the treatment of a variety of diseases. Neutrophils, the most abundant leukocytes in mammalian circulation, are the first type of leukocytes to migrate to the site of inflammation and can be used for cellular drug delivery [13]. The related treatment strategies are divided into two categories. One is based on the design of nanocarriers. In this category, surface-specific modified nanocarriers target activated neutrophils in vivo after intravenous injection. Then, anti-inflammatory or anticancer agents can hijack the neutrophils to cross the vascular barrier and target the inflammatory or tumor site by using the natural characteristics of neutrophils [14–16]. Another strategy is based on the neutrophils themselves, using neutrophils as carriers to load drugs and subsequently injecting them into the body. Neutrophils infiltrate the tumor through the concentration gradient of chemokines, which increases drug accumulation in the lesions and significantly improves the therapeutic effect [17, 18]. In preliminary studies, surgery, radiotherapy (RT), or photothermal therapy (PTT) was used to amplify the inflammatory signal of the tumor microenvironment (TME), and then hitchhiking nanodrugs were delivered to residual lesions through the natural inflammatory chemotaxis of neutrophils. These works provided promising prospects and laid the foundation for exploitation of more combination therapy strategies involving clinically applied therapies and the innate biological functions of neutrophils [17–19]. However, patients suffering from cancers such as unresectable hepatocellular carcinoma (HCC) have limited response to RT and may require multiple rounds of treatment and multiple radiation sessions [20, 21]. Although PTT has been used in clinical practice for more than 40 years to treat a variety of cancers, including superficial skin lesions and esophageal and lung tumors, its therapeutic effect on deep tumors is limited [22]. As the only noninvasive ablation modality, HIFU has been approved by the U.S. Food and Drug Administration (FDA) for use in the treatment of many kinds of solid tumors without limitation of the depth of the tumor site [23]. Furthermore, patients who receive HIFU ablation can avoid surgical trauma and radiation side effects. Therefore, we anticipate that the combination of HIFU and a neutrophil-mediated drug delivery system may be a more practical anticancer strategy than existing strategies.

**Scheme 1:**
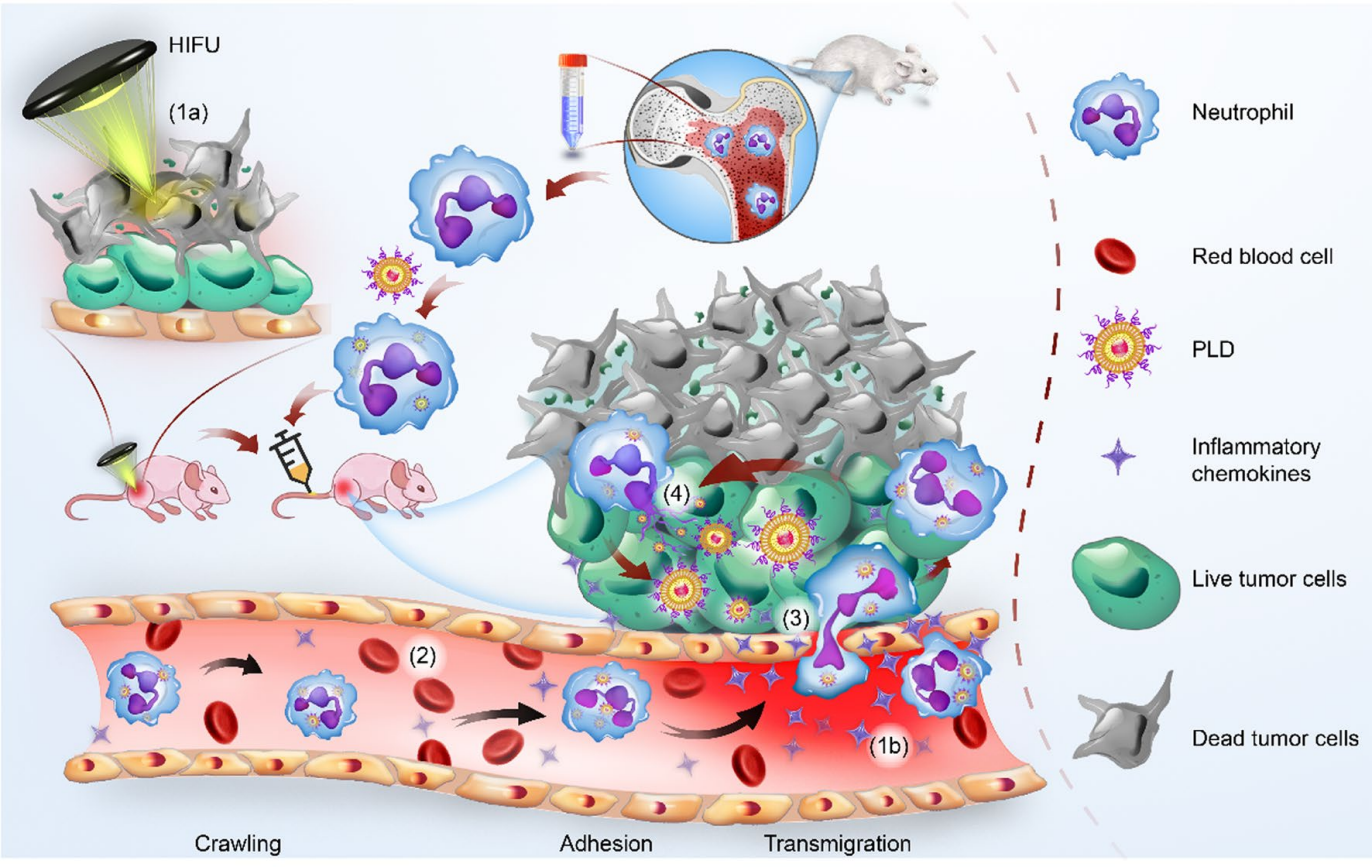
Schematic illustration of neutrophil-mediated chemotherapeutic drug delivery for suppression of tumor recurrence after HIFU ablation. HIFU-induced cell death creates an inflammatory environment in the residual tumor (1a and b), and the released chemokines induce neutrophils to migrate to the tumor site (2). PLD@NEs transmigrate to the vascular barrier and penetrate into the residual tumor along the chemotactic gradient (3). PLD is released from neutrophils to produce an antitumor effect via NET formation (4)

Herein, based on the changes in the TME after HIFU treatment, we developed an innovative strategy of inflammation-tendency neutrophil-mediated clinical nanodrug targeted therapy for residual tumors after HIFU ablation. Neutrophils were used as natural carriers in combination with PEGylated liposome doxorubicin (PLD, the first liposome drug approved by the FDA in 1995), a model chemotherapeutic nanodrug [24, 25], to construct an efficient, innovative cell therapy drug (PLD@NEs). To establish proof of concept, liver cancer was selected as a tumor model, and a neutrophil-mediated drug delivery system (PLD@NEs) was used to inhibit the recurrence of liver cancer after HIFU ablation. This anticancer strategy showed “triple-win” prospects, demonstrating the capacity for (a) direct thermal ablation of the tumor to kill most of the lesion; (b) targeted drug delivery through ablation-induced inflammation; and (c) reduction of the side effects of systemic chemotherapy, which is an urgent problem to be solved in clinical cancer treatment. The in vitro and in vivo results showed that PLD@NEs targeted, infiltrated and accumulated in residual tumor tissue and locally released chemotherapeutic drugs via inflammatory stimulation to treat residual tumors and thereby inhibit tumor recurrence (Scheme 1). Furthermore, because it is a combination of clinically recognized treatments, the proposed strategy has excellent potential for clinical translation.

## Results

### Preparation and characterization of PLD@NEs

**Fig. 1:**
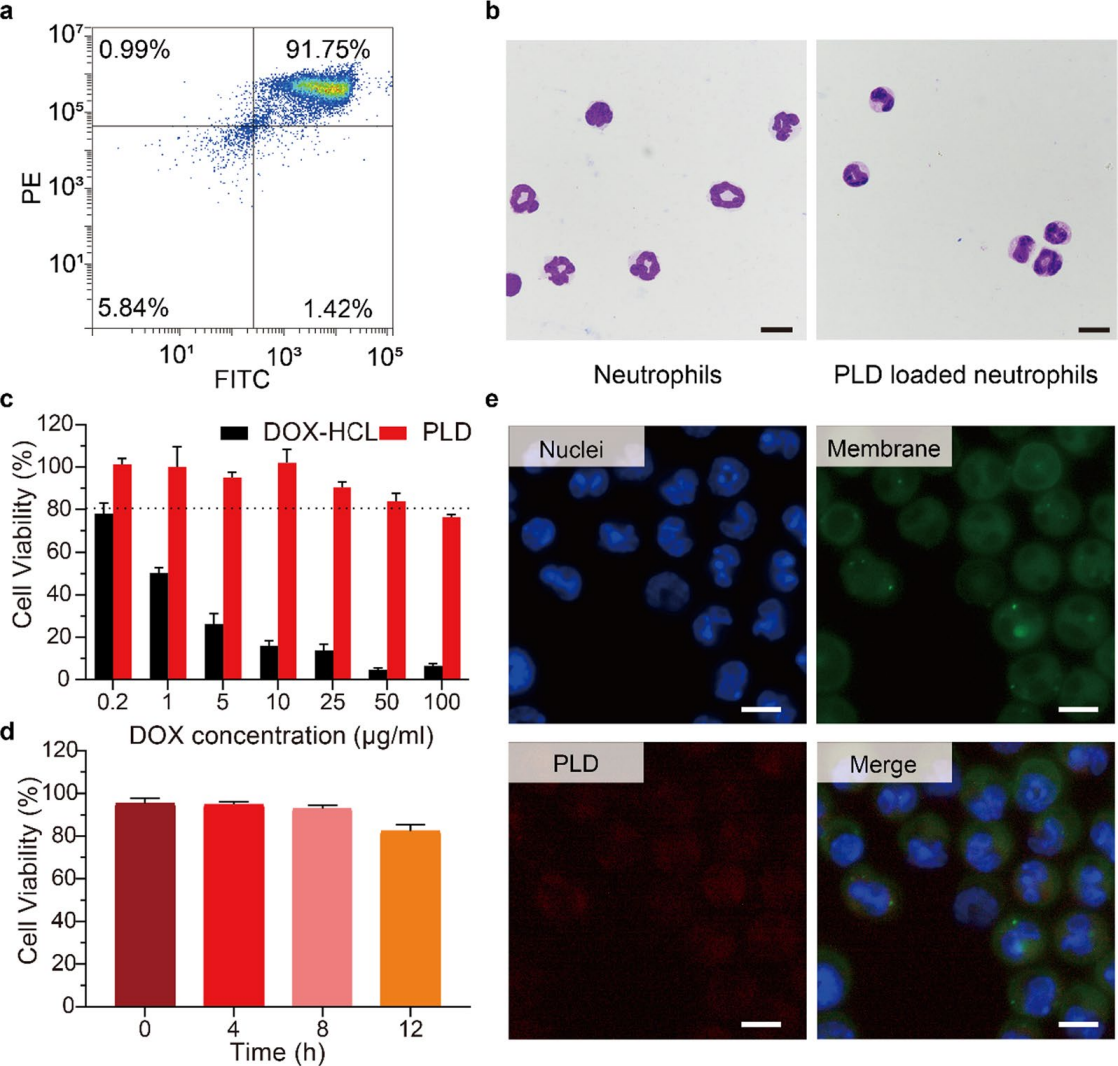
Preparation and characterization of PLD@NEs. **a** Flow cytometry analysis of the purity of isolated neutrophils stained with FITC-conjugated Gr-1 and PE-conjugated CD11b antibodies. The average population of cells is shown in the corner of each quadrant. **b** Morphological images of neutrophils (left) and PLD@NEs (right) stained with Wright-Giemsa stain. Scale bar: 10 μm. **c** Cytotoxicity of DOX and PLD against neutrophils. n = 5. **d** Cell viability of PLD@NEs during 12 h of incubation. n = 5. **e** Fluorescence microscope images of PLD@NEs. The nuclei and membranes of neutrophils were stained with DAPI and DiO, respectively. Scale bar: 10 μm

**Fig. 2:**
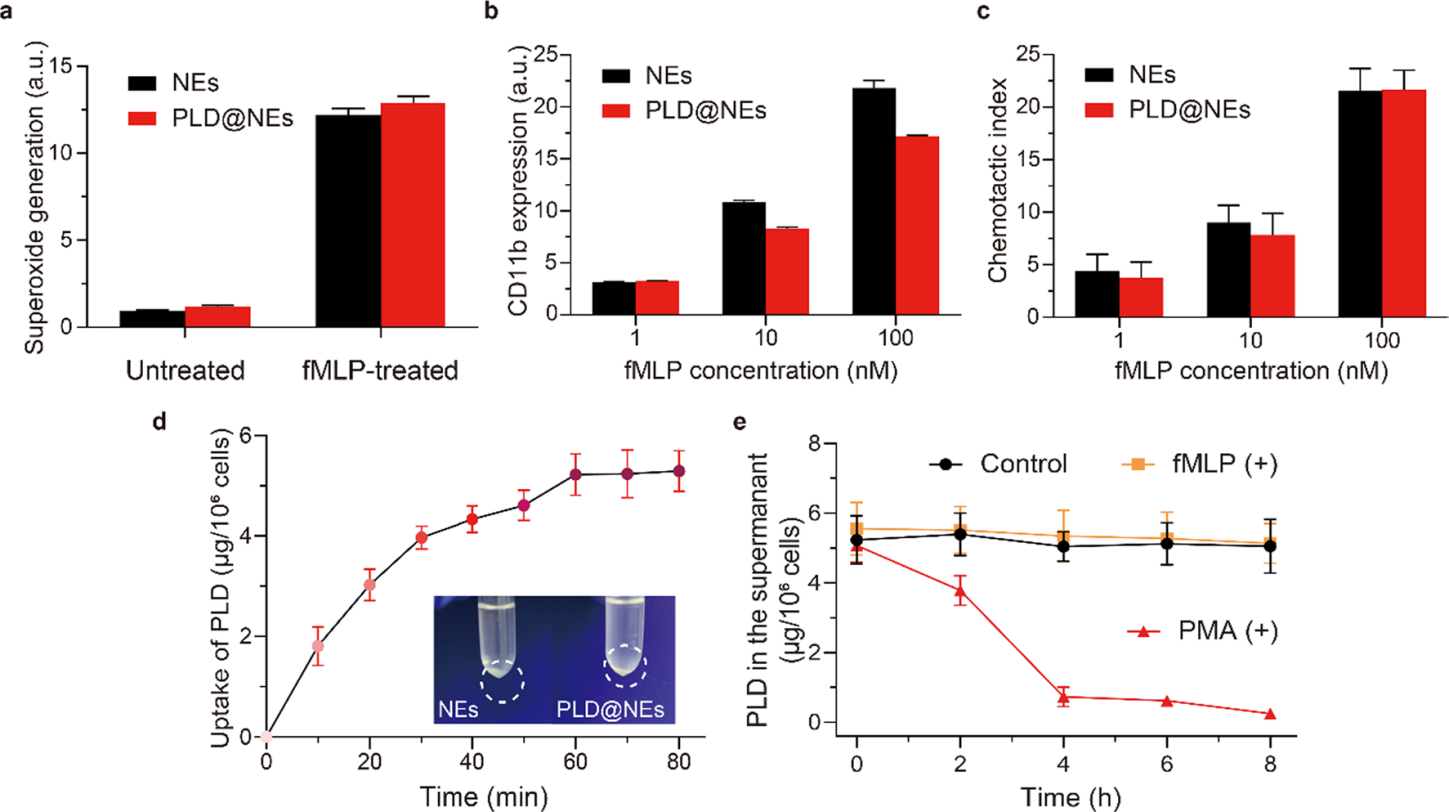
Physiological functions of neutrophils and PLD@NEs under different conditions. **a** Superoxide generation after stimulation with fMLP for 0.5 h. n = 3. **b** Changes in CD11b expression on the membranes of neutrophils after treatment with different concentrations of fMLP for 0.5 h. n = 3. **c** Chemotactic activity after treatment with different concentrations of fMLP for 0.5 h. n = 3. **d** Cellular uptake of PLD by neutrophils (n = 3) and color changes of neutrophils before and after PLD loading. **e** Determination of the amounts of PLD retained in PLD@NEs under the absence of fMLP/PMA, the presence of fMLP (100 nM), or the presence of PMA (100 nM). n = 3

To construct an inflammation response-targeting drug delivery system, mature neutrophils were isolated from the bone marrow of murine hind limb long bones by using a discontinuous three-layer Percoll density gradient as previously described [26]. Based on the determined expression levels of mature mouse neutrophil-specific biomarkers (including Ly6G and CD11b), the purity was quantified as greater than 90% (Fig. 1a). The cells were round and donut-shaped with segmented nuclei, and approximately 8 × 10^6^ cells were obtained per mouse (Fig. 1b). The survival rate of the isolated neutrophils was approximately 98.7% and remained above 90% within 12 h. These results revealed that neutrophils were successfully extracted from mouse bone marrow and that they were of sufficient quantity and quality to be used for drug encapsulation. The cytotoxicity of PLD (Fudan-Zhangjiang) against neutrophils was studied by CCK-8 assay. Within 12 h of incubation, PLD showed no obvious toxicity to neutrophils at the tested concentrations. The control, free doxorubicin (DOX), showed extreme cytotoxicity against neutrophils (Fig. 1c). These results illustrated why we chose a liposomal drug (PLD) as a model drug: because free chemotherapeutic drugs (DOX) directly kill neutrophils in a short time and therefore cannot be used to prepare chemo@NEs. The commercial nanodrug PLD was incubated with purified neutrophils to form PLD@NEs. There was no significant morphological change in neutrophils after PLD loading (Fig. 2b, e). The activity of neutrophils was confirmed 12 h after loading (Fig. 1d and Additional file 1: Figure S1). Neutrophils circulate in the bloodstream with a half-life of approximately 8 h [27]. The results demonstrated that although the proportion of neutrophil death increased with prolonged loading time, the viability was above 80%, proving that PLD@NEs can remain active within the effective time window before migrating to the tumor site.

### Assessment of the physiological functions of PLD@NEs

Considering that the phagocytosis of neutrophils was almost eliminated after 1 h, we chose 1 h as the coincubation time for the preparation of PLD@NEs. The produced PLD@NEs had a loading capacity of approximately 5 µg of PLD per 10^6^ cells, as determined by high-performance liquid chromatography (HPLC) (Fig. 2d). The PLD@NEs were immediately used for further research.

Since the physiological functions of PLD@NEs are essential for their in vivo chemotactic migration, we explored the physiological functions of PLD@NEs in response to inflammatory signals, including their superoxide generation capability, their expression levels of a specific protein (CD11b) and their chemotaxis capability [28, 29]. Formylmethionylleucylphenylalanine (fMLP), a neutrophil chemotactic peptide [30], was used to stimulate PLD@NEs in vitro. As expected, fMLP significantly increased the superoxide levels of PLD@NEs and had an equivalent effect on neutrophils (Fig. 2a and Additional file 1: Figure S2). In addition, the expression levels of CD11b were dramatically increased with stimulation by fMLP in both PLD@NEs and neutrophils (Fig. 2b and Additional file 1: Figure S3). Chemotaxis was investigated with a transwell migration assay, and the corresponding results indicated that PLD did not affect the chemotactic ability of neutrophils (Fig. 2c and Additional file 1: Figure S4). Taken together, these results demonstrate that PLD@NEs maintain the natural physiological functions of neutrophils, which can respond positively to inflammatory signals and migrate to inflammation sites.

Next, the release behavior of PLD from PLD@NEs under three different conditions (normal physiological conditions, chemotaxis process and an inflammatory environment) was explored. To simulate the inflammatory environment in vivo, fMLP and phorbol myristate acetate (PMA) were used as inflammatory factors in the blood circulation and inflammatory sites, respectively [31]. The results showed that most PLD was retained inside neutrophils within 8 h under physiological conditions. Additionally, minimal leakage of PLD from PLD@NEs was detected even after fMLP stimulation for 8 h. In contrast, 87% PLD was released rapidly from PLD@NEs after PMA stimulation (Fig. 2e). This is a prerequisite for in vivo experiments that the rapid response to the inflammatory environment ensures that PLD will be maximally released after reaching the target site.

### Inflammation-directed sequential delivery of PLD@NEs in a 3D tumor spheroid model

**Fig. 3:**
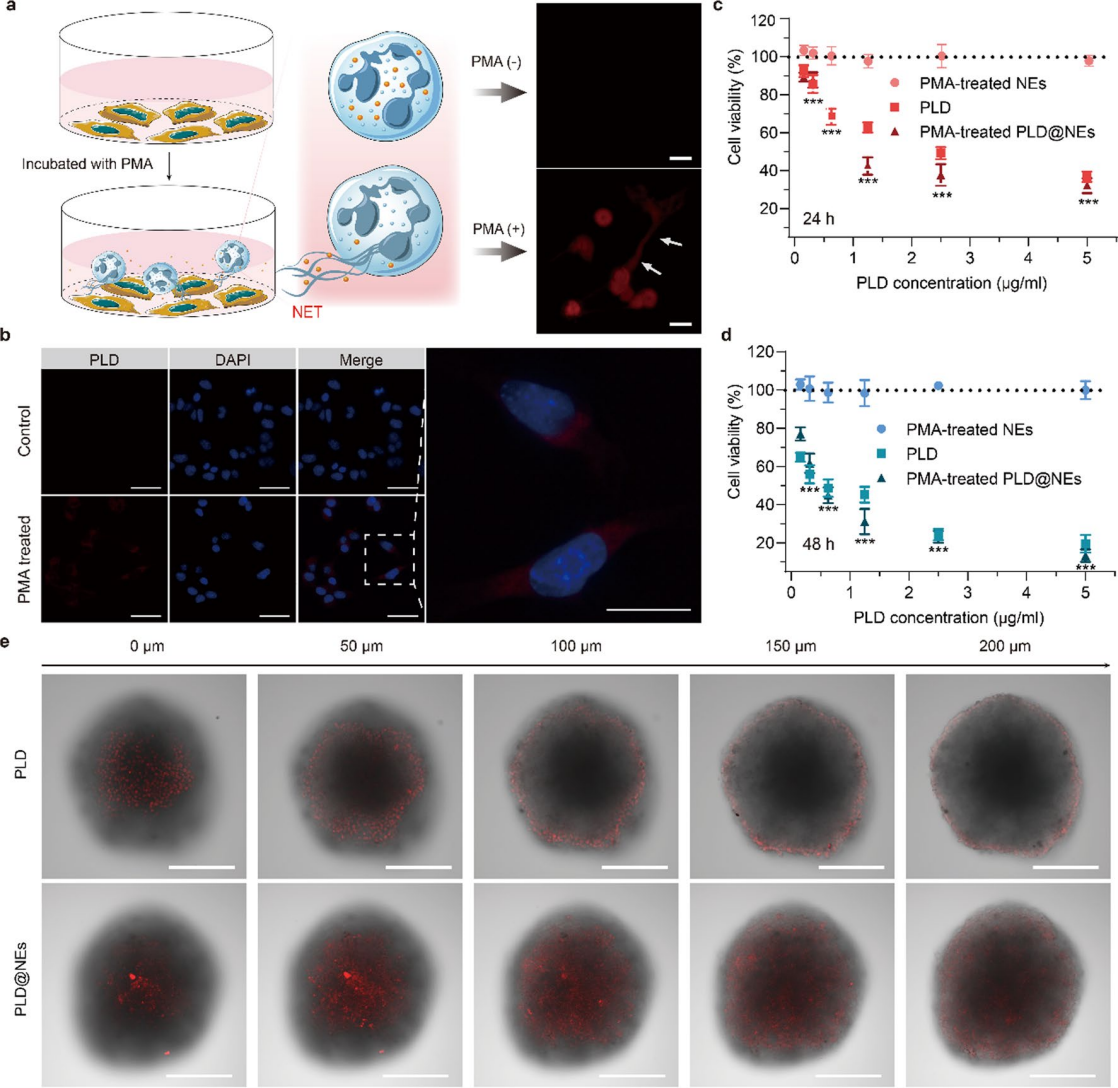
Inflammation-mediated targeting delivery. **a** Schematic illustration of the mechanism of PLD release from PLD@NEs (left). Fluorescence images of NETs released from PLD@NEs after incubation with PMA (100 nM) (upper right) for 4 h in comparison with fMLP (100 nM) (lower right). **b** Fluorescence image of Hepa1-6 cells after incubation with supernatant medium of untreated/PMA (100 nM)-treated PLD@NEs for 8 h. The nuclei were stained with DAPI. Scale bar: 50 μm. **c**, **d** Cytotoxicity of PMA-treated PLD@NEs against Hepa1-6 cells after incubation for 24 h **(c)** or 48 h **(d)**. The data are shown as the mean ± SD. n = 5; *p < 0.05, ** *p*<0.01, *** *p*<0.001. **e** Tumor permeability of PLD@NEs after incubation with 3D Hepa1-6 tumor spheroids for 8 h. CLSM images were obtained from the surface to the middle of the tumor spheroid with a Z-stack thickness of 50 μm. Scale bar: 200 μm

Previous studies have shown that in an inflammatory environment, encapsulated nanodrugs are released from neutrophils through the formation of network structures composed of chromatin and granule protein (known as neutrophil extracellular traps, NETs) [32]. The released nanodrugs are then endocytosed by tumor cells. Thus, we evaluated the formation of NETs in PLD@NEs with PMA stimulation for 4 h (Fig. 3a, left). After PMA stimulation, the red filiform fluorescence of extracellular DNA stained with propidium iodide (PI) was clearly observed in PLD@NEs, indicating the formation of NETs (Fig. 3a, right). These results suggest that it is possible to establish a transport cascade involving neutrophils in which neutrophils release PLD through rupture and the PLD is transferred to target cells (Fig. 3b and Additional file 1: Figure S5).

After proving that PLD can be effectively released from PLD@NEs, we verified the in vitro anticancer effect of PLD@NEs on Hepa1-6 cells using a CCK-8 assay after 24 or 48 h of incubation with the supernatant medium of PLD@NEs that had been treated with 100 nM PMA (Fig. 3c, d). We additionally verified the cytotoxicity of PLD@NEs against HepG2 cells and 4T1 cells (Additional file 1: Figures S6 and S7). Compared with the control, the PLD released from PLD@NEs was equally cytotoxic against tumor cells. The fluorescence images in Fig. 3b and Additional file 1: Figure S5 showed the accumulation of PLD in the cytoplasm after 8 h of endocytosis by tumor cells. Given these findings, it is reasonable to believe that cell-based chemotherapeutics have the capacity to accumulate and release drugs at the tumor site as long as HIFU has primed inflammatory stimulation. These results suggest that PLD can be released from neutrophils under inflammatory conditions and exert a toxic effect on tumor cells that is probably mediated by NET formation.

The penetration depth of nanodrugs in tumors is an important factor affecting the therapeutic efficacy of these drugs against cancers [33]. Based on the verified cytotoxicity of the inflammatory cell-based chemotherapy system, we established Hepa1-6 multicellular spheroids to mimic the TME in vivo [34] and investigated the tumor penetration ability of PLD@NEs. Three-dimensional (3D) liver tumor spheroids with an average size of 400~500 μm were obtained on day 14 post seeding. The levels of the cytokine CXCL1/KC were markedly increased inside spheroids, and a concentration gradient was established between the inside and outside (supernatant medium) of the tumor spheres during growth monitoring (Additional file 1: Figure S8). The formation of this inflammatory factor concentration gradient is a necessary condition for neutrophils to infiltrate into spheroids. Then, we incubated the 3D tumor spheroids with PLD@NEs and detected the DOX signal inside the tumor models to verify the tumor permeability of PLD@NEs. Semi-quantitative analysis showed significant differences in fluorescence intensity between the two groups (Additional file 1: Figure S9). The red fluorescence representing DOX was distributed in most areas of the spheres after 8 h of incubation in the PLD@NE group. However, the DOX signal could only be observed on the periphery of the spheroids in the PLD group (Fig. 3e and Additional file 1: Figure S10). These results suggested that cell-based chemotherapy showed satisfactory tumor spheroid permeability. The results of the in vitro experiment provided a basis for exploring the permeability of PLD@NEs in tumors in vivo.

### HIFU ablation in Hepa1-6 tumor-bearing mice

HCC accounts for approximately 80% of primary liver cancer case and is the most lethal form of liver cancer. Its prevalence rate and incidence rate are increasing year by year. The poor prognosis of patients largely results from the fact that diagnosis of the disease usually occurs at an advanced stage, at which point the disease fails to meet the criteria for operation or transplantation [21, 35]. With the development of technology, HIFU has emerged as an effective image-guided, noninvasive therapeutic modality for multiple solid tumors, including unresectable HCC [36]. However, the main limitation and challenge of HIFU, similar to those of other thermal ablation treatments, is the risk of local recurrence caused by residual tumor tissue after ablation [9, 37]. Notably, sorafenib, the first-line chemotherapeutic drug for HCC, provides unsatisfactory efficacy in patients due to its marginal improvement in the survival rate [38]. Until now, neither ablation nor chemotherapy has effectively improved the prognosis of this devastating disease. We therefore chose liver cancer as a tumor model to explore the effectiveness of the proposed HIFU-cytopharmaceutical chemotherapy strategy.

**Fig. 4:**
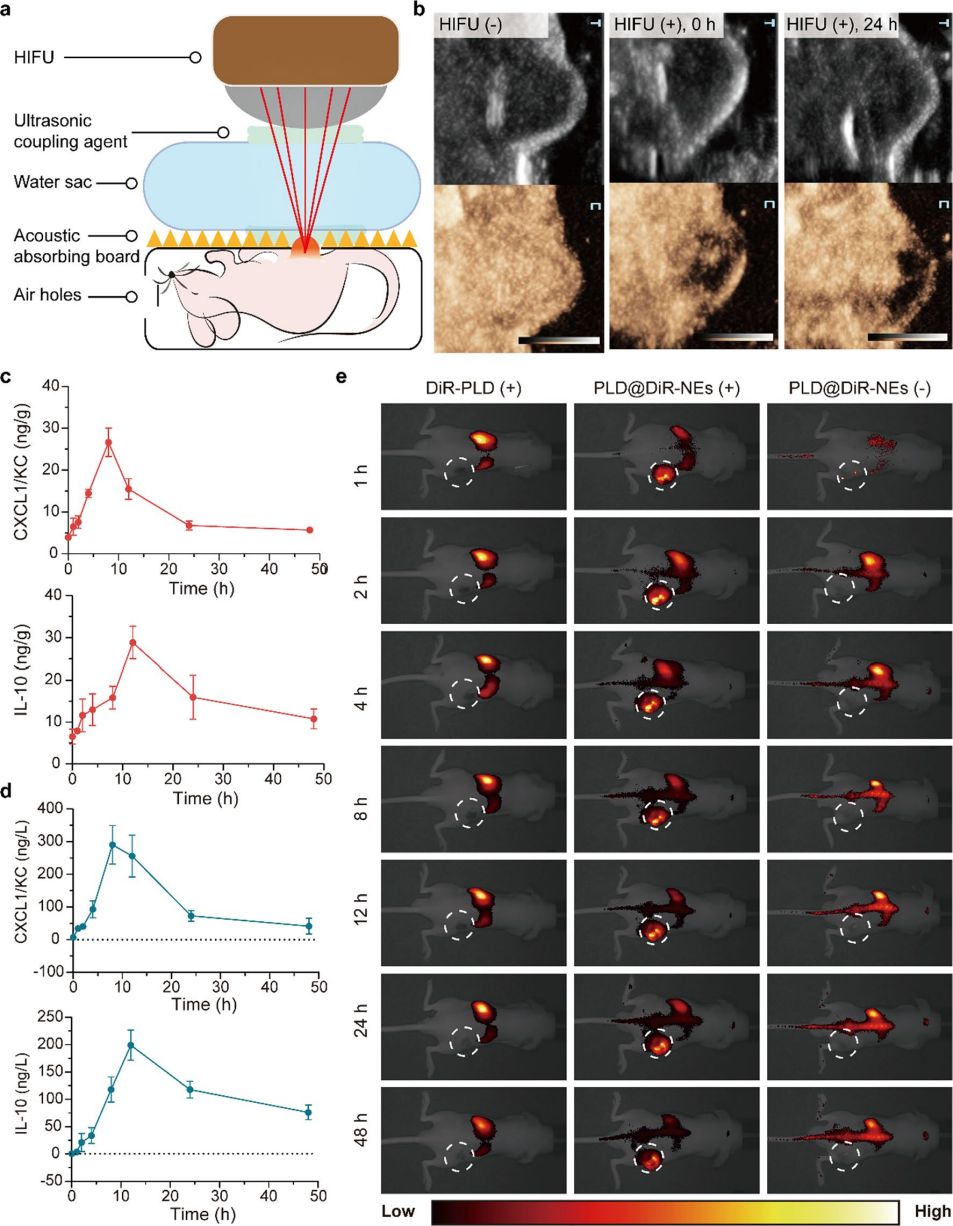
HIFU ablation induced PLD@NE migration to the tumor site. **a** Schematic illustration of HIFU ablation in tumor-bearing mice. **b** Ablation efficiency verified by CEUS in real time. Before HIFU, the perfusion of the tumor was enhanced overall, suggesting that it was a solid tumor with abundant blood flow. After HIFU, a perfusion defect area appeared in the tumor, indicating ablation damage. **c**, **d** Expression levels of CXCL1/KC and IL-10 in tumors (**c**) and serum (**d**) of mice over 48 h after HIFU ablation. n = 3. **e** In vivo fluorescence images of tumor-bearing mice after intravenous administration of DiR-PLD or PLD@DiR-NEs over time. Mice were treated with (−) or without (+) HIFU ablation. PLD@DiR-NEs were injected at a dose of 3 × 10^6^ cells per mouse. The tumor site is indicated by the dashed circle. DiR-PLD, DiR labeled PLD; DiR-NEs, DiR labeled neutrophils

Thus far, HIFU systems based on a single spherical focusing transducer have been widely used in small-animal research [39]. The intensity ranges from 5 to 40 W, which is far lower than that of clinical HIFU devices [40–42]. The HY2900 we used in this study has been used for clinical applications and has a maximum power of 479.2 W; therefore, it was necessary to explore an appropriate ablation method for tumor-bearing mice before the experiment. To treat tumor-bearing mice with HIFU successfully, we made a HIFU box that was suitable for immobilization of the mice and protection of the mice from excessive ablation damage (Fig. 4a). The framework prevented the force of the large water sac from acting directly on the mice and reduced mouse mortality caused by stress. In addition, the surface was covered with an acoustic absorbing board featuring a hole on the surface that allowed only the tumor to be exposed to the acoustic beam; this board effectively controlled the ablation range of the HIFU instrument and protected mice from death caused by overtreatment. The mice tolerated the operation with a low mortality rate (< 1%) and no normal tissue damage after ablation.

B-mode ultrasound showed that the change in the tumor grayscale value during HIFU was not as obvious as the changes in previous studies on rabbits or rats [43, 44]. Contrast-enhanced ultrasound (CEUS) was used to show the blood perfusion of a tumor before and after ablation. Before ablation, the tumor showed homogenous enhancement and a solid appearance. CEUS showed that there were perfusion defects in the tumor, suggesting that HIFU caused partial vascular occlusion (Fig. 4b and Additional file 2: Movie S1). To further confirm the effectiveness, mice were sacrificed on day 0 and day 14, and tumors were dissected. The excised tumors swelled with hemorrhage on day 0 and showed distinct coagulative necrosis on day 14 (Additional file 1: Figure S11). The results of CEUS and pathology showed that HIFU ablation was successfully achieved in tumor-bearing mice.

### Chemotaxis of PLD@NEs to HIFU-ablated tumors

It has been reported that live neutrophils can efficiently target primary tumors [16, 45]. We investigated whether local HIFU could induce enhancement of inflammatory signals in the tumor site and improve the tumor-targeting ability of neutrophils in vivo, thus turning the weakness of HIFU-induced inflammation into advantages. We examined the expression levels of inflammatory cytokines in the tumors and serum of mice after HIFU treatment. CXCL1/KC is a mouse homolog of the proinflammatory cytokine IL-8, which has strong neutrophil chemotactic activity equivalent to that of human IL-8 and can broadcast the locations of residual tumors to neutrophils [46]. As shown in Fig. 4c and d, HIFU constructed an inflammatory TME with concentration gradients of the chemokines CXCL1/KC and IL-10, which was attributable to the proinflammatory response caused by the necrosis of tumor cells and the release of necrotic debris [47]. Such increases in chemokines effectively elevate the accumulation of neutrophils because these cells can actively home to inflammatory sites, exudate from the blood vessels, and penetrate into tumors with the gradients of chemokines.

Next, we labeled PLD and the constructed PLD@NEs with the fluorescent dye 1,1AQ1′-dioctadecyl-3,3,3′,3′-tetramethylindotricarbocyanine iodide (DiR) to investigate chemotactic migration in vivo with an in vivo imaging system (IVIS). In the PLD@NE group, there was a significantly stronger capability of residual tumor targeting in the group of HIFU-treated tumor-bearing mice than in the other groups (Fig. 4e). A fluorescent signal was observed at the tumor site 1 h post injection in the PLD@NE (+) group and was maintained for at least 48 h during our observation. The results prove that tumor tissue can elicit a sufficient inflammatory response to facilitate chemotactic migration of PLD@NEs to the residual tumor site.

### In vivo therapeutic efficacy and biosafety evaluation of PLD@NEs in HIFU-treated tumor-bearing mice

**Fig. 5:**
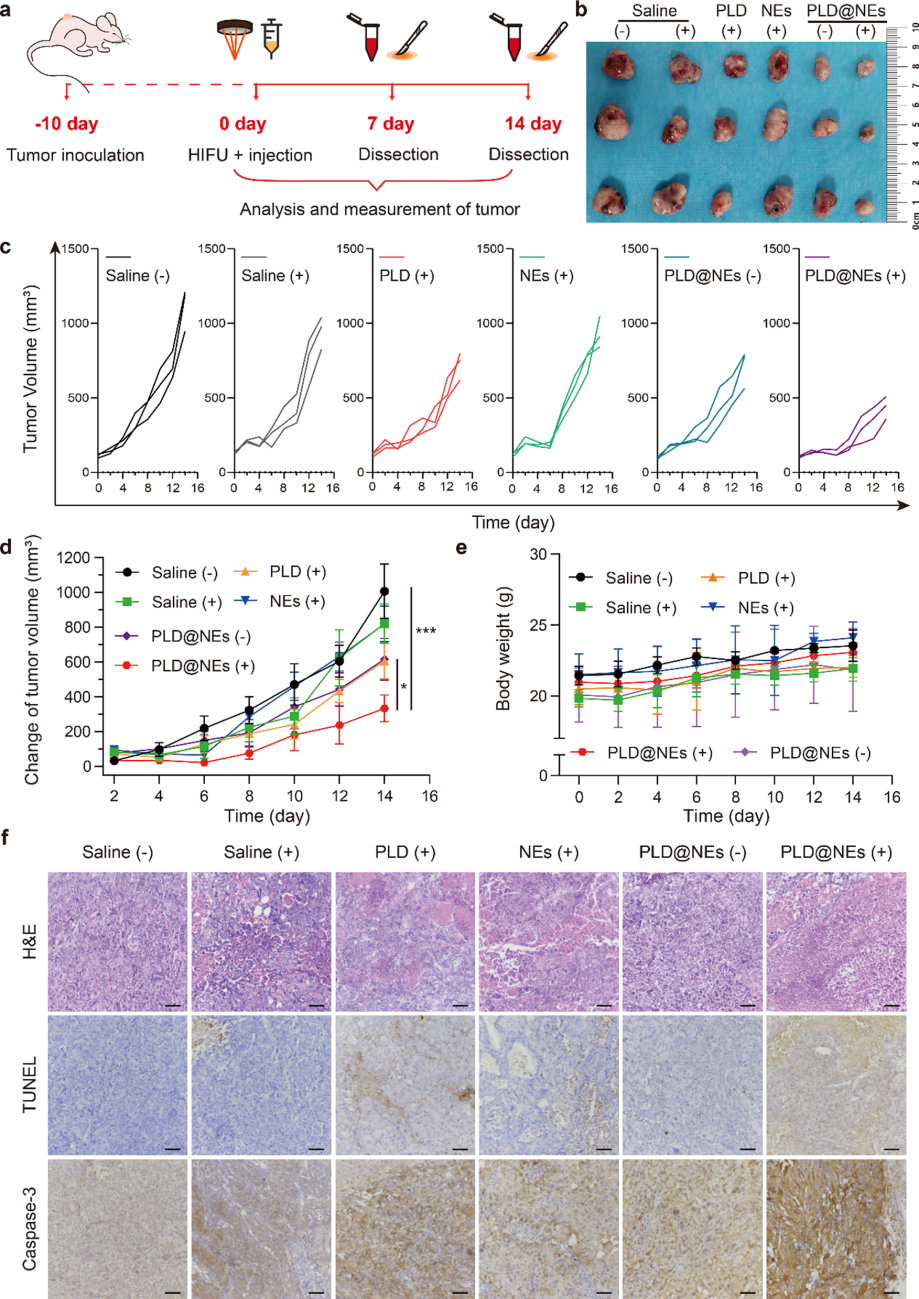
Therapeutic effect mediated by HIFU ablation and neutrophil-based chemotherapy. **a** Schematic illustration of neutrophil-mediated active chemotherapeutic drug delivery for suppression of hepatoma recurrence after HIFU. Mice were treated with (+) or without (-) HIFU and subsequently received different formulations of saline, PLD, blank neutrophils (NEs), or PLD@NEs. Blood and tumors were harvested at day 7 and day 14 for biotoxicity evaluation. **b** Representative images of isolated Hepa1-6 tumors after different treatments on day 14. n = 3. **c** Individual tumor growth kinetics of each group. n = 3. **d** Mean percentage change in tumor volume relative to the initial volume. n = 3; * *p *< 0.05, ** *p *< 0.01, ***p < 0.001. **e** The body weight changes of tumor-bearing mice were monitored during the administration of treatment. **f** Histological observation of tumors excised from hepatoma-bearing mice after treatment at day 14. The tumor sections were stained with H&E (top row), TUNEL (middle row) and a caspase-3 antibody (bottom row). Scale bar: 50 μm

Encouraged by the in vitro results, we finally evaluated the therapeutic efficacy of PLD@NEs in a HIFU-treated HCC-bearing mouse model. After treatment with individualized HIFU ablation, PLD@NEs were subsequently administered intravenously to mice at a dose of 5 × 10^6^ cells per mouse (equivalent to 25 µg of DOX) (Fig. 5a). In clinical practice, incomplete ablation occurs when the treatment area is limited to reduce damage to normal tissue around lesions or to accommodate unique tumor locations (e.g., liver cancer in the diaphragm area); this is exactly the problem we are trying to solve. As shown in Fig. 5b–d, ablation with HIFU alone suppressed tumor growth within 4 days. However, tumor regeneration was observed, as incomplete ablation only induced part of the tumor to undergo apoptosis and necrosis. In contrast, the PLD@NE (+) group displayed potent tumor inhibition compared to that of all other groups, including the PLD (+) group (Fig. 5d). The results suggested that the increased recruitment of PLD@NEs to the tumor site enhanced the effectiveness of chemotherapy. In addition, no significant inhibitory effects were observed in the neutrophil group and the saline group, suggesting that the neutrophils lacked antitumor capacity and did not interfere with treatment efficacy. Under the upregulation of inflammatory factors induced by HIFU, PLD@NEs broke down their nuclear contents and released NETs, which facilitated the release of PLD from the neutrophils to kill tumor cells [48]. We also utilized histological analysis to observe the cell morphology in the recurrent tumors. The tumors collected from the PLD@NE (+) group showed massive tumor cell death. Furthermore, apparent caspase-3 activation was observed in the PLD@NE (+) group compared with the other groups. A TUNEL assay also revealed increased apoptosis in the PLD@NE group (Fig. 5f). The systemic toxicity of PLD@NEs was evaluated by monitoring the body weight of the mice and performing a blood biochemical assay after treatment. As shown in Fig. 5e, no body weight loss was identified following treatment with PLD@NEs (+), and the serum liver enzyme levels of mice in the PLD@NE (+) group were similar to those of mice in the saline group (Additional file 1: Figure S13), suggesting that PLD@NEs (+) have no serious systemic toxicity. Moreover, major organs were excised from mice on day 14, and no pathological manifestations were observed in these organs after treatment with PLD@NEs (Additional file 1: Figure S12).

Taken together, these data provide evidence that the therapeutic strategy combining HIFU with neutrophil-mediated chemotherapy exhibited good tumor suppression efficacy, showing potential for future application in clinical situations.

## Discussion

Here, we developed a neutrophil-mediated targeting nanodrug to establish an efficient strategy for the treatment of residual tumors after HIFU ablation. Our approach includes several consecutive steps (Scheme 1). (1) HIFU ablation creates an inflammatory environment in the tumor site, stimulating the transmigration of neutrophils. (2) Neutrophils carry the chemotherapeutic drug PLD (PLD@NEs) to the tumor site, exude the endothelial lining of blood vessels, and penetrate into the tumor, thus overcoming the biological barriers encountered by traditional drug delivery approaches. (3) Once PLD@NEs enter tumor tissue, PLD is released from the PLD@NEs through the reticular structure and then internalized by tumor cells. This administration strategy endows the clinical drug PLD with favorable tumor-targeting capacity and permeability and an ability to exert antitumor effects at a dose lower than the recommended dose.

Nanotechnology has made immense progress in the last decade and provides a novel perspective for last-resort treatments for refractory malignancies. Despite efforts to optimize the physicochemical properties of nanocarriers, such as their size, shape or binding affinity, some limitations continue to hinder the application of nanomaterials in drug delivery [49]. Moreover, the extensive heterogeneity of drug delivery systems with regard to patients and tumors remains a barrier to efficacy and clinical translation [50]. The tumor vascular system also restricts drug access to deep tumor tissues, especially in solid tumors [51]. We are committed to building an alternative strategy to integrate and improve the existing clinical therapies rather than focusing on new engineered nanotherapies. Previous studies have confirmed the feasibility of neutrophil-based targeted drug delivery systems for a variety of disease; these systems have shown good capabilities for active targeting and passage across vascular barriers (e.g., the blood–brain barrier) [17]. In our research, we prepared 3D tumor spheroids with concentration gradients of inflammatory factors inside and outside the spheroids to mimic the TME (Additional file 1: Figure S8). Further results showed that drug-loaded neutrophils were able to deliver the agent inside the tumors (Fig. 3e) and the delivery efficiency of the drug after being carried by neutrophils has roughly doubled according to semi-quantitative analysis (Additional file 1: Figure S9). Before administering cytopharmaceutical chemotherapy, traditional therapy, such as surgery, PTT or RT, was performed to establish therapy-associated inflammation in order to activate the neutrophil-mediated drug delivery system [17–19]. HIFU, the only noninvasive ablation therapy for deep tumors, has not yet been explored in this combination therapy.

Currently, focused ultrasound is approved by the FDA for the clinical treatment of prostate cancer, Parkinson’s disease and bone metastasis. Meanwhile, the application of HIFU for the treatment of breast cancer, pancreatic cancer, renal cell carcinoma, liver cancer, soft tissue tumors and other malignant tumors has also been explored (Focused Ultrasound Foundation: http://www.fusfoundation.org). Many of the early large-sample clinical trials were conducted by Chinese researchers. For example, Feng et al. described clinical experience involving 1038 patients with various kinds of solid tumors treated with HIFU. A 4-year follow-up confirmed the safety and effectiveness of HIFU treatment for multiple carcinomas [8]. In recent years, with technological advancement, HIFU-related clinical trials have been carried out worldwide (NCT04796220, NCT04852367, NCT04573881). HIFU has become a promising alternative treatment to conventional surgical resection or enhanced adjuvant therapies (such as chemotherapy, RT or immunotherapy) [9]. Our results showed that the inflammatory response in the TME after HIFU ablation was sufficient to attract neutrophils to migrate to the tumor site and rapidly release the drug (Fig. 4e). Although it has been reported that neutrophils might participate in cancer metastasis by protecting and transporting cancer cells following cell-cell interactions [52], our strategy solves this problem. On the one hand, PLD@NEs can be recruited to the inflammatory microenvironment of residual cancer in situ and release drugs to kill residual cancer cells. On the other hand, the escaped cancer cells can be “surrounded” and killed by the released drugs after neutrophil death. This is reason we believe that our strategy turns the disadvantages of HIFU treatment into advantages. In addition, we have also considered this problem, so we set up HIFU (+), NEs group in the experimental group. The result showed there is no significantly difference between HIFU (+), NEs group and HIFU (+), Saline group (Fig. 5d). We think it might be because the number of NEs injected is limited, which is not enough to have a significant impact on tumor progression. Therefore, our proposed neutrophil-based combined therapy strategy is of great significance for the treatment of unresectable tumors, chemotherapy/RT-resistant tumors or deep tumors.

To the best of our knowledge, four types of PLD are available clinically: Doxil, Doxisome, Lipo-Dox, and LIBOD [53]. The main difference among them lies in the composition or proportions of synthetic lipids. We used PLD as a model cytochemotherapeutic drug because the free chemotherapeutic drugs kill neutrophils, which would have made it impossible to load the drugs directly into neutrophils for further use. However, the nanodrug showed good biocompatibility and low cytotoxicity during incubation with neutrophils (Fig. 2c, d and Additional file 1: Figure S1). In addition, recent research has revealed that anti-PEG antibodies are present in many healthy individuals as well as in patients receiving PEG-functionalized drugs, which can result in rapid release of encapsulated DOX from liposomes. This procedure may alter the therapeutic efficacy and safety in patients with high levels of pre-existing antibodies against PEG [53]. In our research, we avoided this problem by loading PLD into neutrophils. Therefore, more anticancer drug can accumulate in the tumor site with the increase in the cytokine concentration gradient.

Although our data suggest a promising approach for hepatoma therapy, there are still several limitations. Neither 3D tumor spheroids nor subcutaneous xenograft tumors can necessarily reflect the real TME in spontaneously occurring cancer [54]. We will explore the construction of tumor organoids and patient-derived organoid transplantation (PDOX) in further research [55]. In addition, more convincing evidence of efficacy and safety is needed to achieve clinical translation, and the feasibility of batch extraction of enough human neutrophils for treatment needs to be determined. However, autologous bone marrow transplantation (ABMT) is a well-known and mature technique. Our plan is to further extract and purify neutrophils from bone marrow and use them to encapsulate drugs. HIFU (a clinical technique for tumor ablation) and PLD (an anticancer chemotherapeutic drug) have both been approved by the FDA, but how to maintain the activity of isolated neutrophils and accurately calculate the dose of delivered drug are challenges that must still be addressed to enable clinical translation.

## Conclusions

This study suggests that HIFU ablation combined with a neutrophil-mediated drug delivery system is a promising therapeutic approach for anticancer treatment. We focused on therapy-induced inflammation to explore a treatment strategy that can transform a weakness into a strength. Our strategy enhanced the accumulation of the chemotherapeutic drug in tumors, maximized the anticancer effects by taking advantage of both methods, and overcame the weaknesses of HIFU and chemotherapy. It therefore shows “triple-win” prospects. First, direct thermal ablation of the tumor is performed to kill most of the cells in the lesion (tumor size is one of the indications for surgical treatment). Second, the ablation-induced inflammation is used to achieve active targeting (the enhanced permeability and retention [EPR] mechanism cited in preclinical studies has not been effectively verified in the clinic). Third, the strategy reduces the side effects of systemic chemotherapy (targeted accumulation can reduce the dose of chemotherapeutic drug needed). These advantages address problems that urgently need to be solved in clinical cancer treatment. In general, our neutrophil-mediated drug delivery system could be an impressive way to integrate smart nanoparticle design with HIFU in cancer therapy. This strategy effectively bridges the two clinical treatment methods, shedding a new light for clinical translation.

## Materials and methods

### Cell culture

Hepa1-6 and HepG2 cells were purchased from the Stem Cell Bank of the Chinese Academy of Sciences. 4T1 cells were kindly provided by Dr. Xue Xie (Shanghai Jiao Tong University Affiliated Sixth People’s Hospital). The Hepa1-6 cells were cultured in high-glucose DMEM. The 4T1 cells were cultured in 1640 medium. The HepG2 cells were cultured in MEM. All the media were supplemented with FBS (10%, v:v), penicillin (100 U/ml) and streptomycin (100 µg/ml). All the cells were cultured at 37 °C in a humidified environment containing 5% CO_2_.

Ex vitro 3D tumor spheroids of Hepa1-6 cells were obtained using a liquid overlay method. Each well of 96-well plates was precoated with 100 µl of FBS-free medium containing sterile agarose (1.5%, w:v). Subsequently, Hepa1-6 cells (5000 cells) were seeded into each well and cultured in complete medium containing FBS (10%, v:v). The tumor spheroids were harvested on day 14, and the average diameter was measured to be approximately 400~500 μm. The formation of hepatoma spheroids was monitored by optical microscopy (Nikon, Japan).

### Preparation and characterization of PLD@NEs

PLD@NEs were obtained by incubating PLD (Fudan-Zhangjiang, Shanghai) with neutrophils. Freshly isolated neutrophils were cultured in sterile tubes with different concentrations of PLD at 37 °C in a humidified environment containing 5% CO_2_. After washing with PBS three times, PLD@NEs were obtained and immediately used for subsequent research. The amounts of DOX loaded in the neutrophils were quantified by HPLC (Agilent). Cell lysis buffer (Beyotime) was added to the PLD@NEs to disrupt the cells and induce PLD release from the cells. Then, the cell lysate was centrifuged at 10,000×*g* for 5 min. Afterward, the supernatant (100 µl) was collected and mixed with 400 µl of methanol. The mixture was vortexed for 5 min and centrifuged at 15,000×*g* for 5 min. The supernatant (20 µl) was injected into the HPLC system for quantification. The morphology of loaded neutrophils was observed using the same method as that previously mentioned.

### Evaluation of physiological functions

To achieve inflammation-targeted drug delivery, the physiological functions of PLD@NEs were evaluated, including the inflammation-responsive expression of the specific cytokine CD11b, chemotaxis and superoxide anion production. The inflammation-mediated superoxide generation capability of PLD@NEs was determined with dihydroethidium (DHE, Beyotime). Neutrophils or PLD@NEs (1 × 10^5^ cells) were incubated with fMLP (1 µM) at 37 °C for 30 min, washed with PBS three times and stained with DHE (5 µM) at 37 °C for 30 min. The fluorescence intensity was measured by flow cytometry (Beckman).

The chemotaxis of PLD@NEs was investigated by transwell migration assay (transwell polycarbonate membrane: 3 μm pore size, 6.5 mm diameter and 0.33 cm^2^ membrane surface area, Corning). Blank neutrophils and PLD@NEs (2 × 10^5^ cells) were added to the upper chamber of the Transwell plate. The lower chamber of the Transwell plate was filled with 600 µl of FBS-free culture medium containing different concentrations of fMLP. After 30 min of incubation, the cells inside the upper chamber were eliminated and centrifuged at 2500 rpm for 5 min to harvest the migrating cells. The chemotaxis index was calculated as (N_fMLP_-N_control_)/N_control_, where N_fMLP_ or N_control_ refers to the number of neutrophils in the lower chamber under incubation conditions with or without fMLP, respectively.

The expression level of CD11b on the membrane was examined by flow cytometry. Neutrophils and PLD@NEs were incubated with different concentrations of fMLP at 37 °C for 30 min. After washing with PBS three times, the cells were incubated with a FITC-conjugated CD11b antibody (1 µg/ml, BioLegend) for 30 min. After another cycle of PBS washing, the fluorescence intensity was determined.

### Cytotoxicity of PMA-treated PLD@NEs against tumor cells

The in vitro cytotoxicity of PLD@NEs after PMA stimulation against Hepa1-6 cells was determined by CCK-8 assay. Hepa1-6 cells (1 × 10^4^ cells) were seeded in a 96-well plate and cultured for 24 h. PLD@NEs were prepared and pretreated with PMA (100 nM) for 4 h. Then, both untreated and PMA-treated PLD@NEs were centrifuged at 1500 rpm for 5 min. The supernatant was obtained and incubated with Hepa1-6 cells for different times. Afterward, CCK-8 solution was added to a final concentration of 10 % (v:v). The absorbance was measured at a test wavelength of 450 nm by a microplate reader (Thermo Fisher). The cell viability was calculated as (A_sample_-A_blank_)/(A_control_-A_blank_) ×100%. The cytotoxicity of PLD@NEs against HepG2 and 4T1 cells was assessed using the same methods.

### Inflammation-mediated drug permeability in 3D tumor spheroids

The in vitro drug delivery capability was determined by assessing the drug permeability depth of PLD@NEs in tumor spheroids. The 3D tumor spheroids were cultured and harvested as previously mentioned. At predetermined time intervals, the culture medium was sampled, and the tumor spheroids were gently dispersed into single cells and centrifuged at 400×*g* for 5 min. The levels of the cytokine CXCL1/KC were detected for 14 days with corresponding enzyme-linked immunosorbent assay (ELISA) kits according to the manufacturer’s instructions. The tumor spheroids were then transferred to a confocal dish and incubated with 1 ml of PLD@NEs (1 × 10^6^ cells, equivalent to 5 µg of PLD) or PLD for 8 h. Images were obtained in real time at a fixed depth of 50 μm from the surface to the middle of the tumor spheroid using Z-stack tomoscanning (Olympus, Japan).

### Mice and ectopic liver cancer model establishment

All animals were treated in accordance with the Guide for the Care and Use of Laboratory Animals, and the protocols were approved by the Animal Experimentation Ethics Committee of Shanghai Jiao Tong University Affiliated Sixth People’s Hospital. The Institute of Cancer Research mice (BALB/c, male, 6 to 8 weeks old, and nude mice, male, four weeks old) were provided by the Animal Laboratory of Shanghai Jiao Tong University Affiliated Sixth People’s Hospital (No. DWLL2021-0677).

To establish a heterotopic tumor model, each mouse was given a percutaneous injection of Hepa1-6 cells (2 × 10^6^ cells per mouse). The status of tumor growth was monitored daily. When the tumor size measured approximately 100 mm^3^, the mice were treated according to the protocol. The tumor volume was calculated as length×width^2^/2.

### HIFU ablation therapy

An HY2900 HIFU system (Wuxi Haiying Technology, Wuxi, China) was used in this study. A diagnostic transducer was localized in the center of the therapeutic transducer. The frequencies of the diagnostic transducer and therapeutic transducer were 3.5 MHz and 1.5 MHz, respectively. The focal region of the therapeutic transducers was an ellipsoid region with dimensions of 8 mm along the beam axis and 1.15 mm in the transverse direction, which was calibrated using a PVDF needle hydrophone with a spot diameter of 0.5 mm in a tank filled with degassed water. To optimize the survival rate of tumor-bearing mice that underwent HIFU, we designed a flexible box to perform the treatment (Fig. 4a). Due to the high energy produced by HIFU, we placed an additional water sac between the matrix and transducer. For acoustic coupling, conventional ultrasound gel was applied. The focal spot of HIFU was located at the lower center of the solid tumor. Considering that the tumor was small, treatment was performed in a horizontal point-by-point mode with one layer. Voltage was applied at 5 V, the pulse duration was 1000 ms, and the exposure separation was 3000 msec between points. The interval distance between points was approximately 1 mm. B-mode ultrasound was used to guide HIFU therapy and monitored the entire process of treatment in real time. The therapeutic efficiency was determined using CEUS and histology.

### Statistical analysis

All experiments were repeated at least three times, and each condition was analyzed in triplicate. Animals were randomly selected for different groups prior to the initiation of the treatment, but the groupings were not blinded for outcome assessment and data analysis. Statistical analysis was performed by using Prism 8.0 (GraphPad) and Excel (Microsoft). All data are expressed as the means ± SDs, as indicated. For comparisons between two groups, two-tailed Student’s t-tests were applied. For comparisons among more than two groups, ANOVA with multiple comparison adjustments was applied. Statistical significance was denoted by asterisks in the appropriate figures (defined as *p < 0.05, **p < 0.01, ***p < 0*.*001).

## Supplementary Information

**Additional file 1.** Supporting information.

**Additional file 2.** Movie S1.

## Data Availability

All data generated or analyzed during this study are included in this published article and its additional files.

## Abbreviations

HIFU: High-intensity focused ultrasound
PLD: PEGylated liposome doxorubicin
TME: Tumor microenvironment
DOX: Doxorubicin
NE: Neutrophil
fMLP: Formylmethionylleucylphenylalanine
PMA: Phorbol myristate acetate
HCC: Hepatocellular carcinoma
NET: Neutrophil extracellular trap
CEUS: Contrast-enhanced ultrasound
