# Supplementary material for: Neutrophil-mediated clinical nanodrug for treatment of residual tumor after focused ultrasound ablation
Source: J Nanobiotechnology. 2021 Oct 29;19:345. doi: 10.1186/s12951-021-01087-w (PMC8555249; doi:10.1186/s12951-021-01087-w)
Supplement: Supplementary file 1 — Additional file 1. Supporting information. [file 12951_2021_1087_MOESM1_ESM.docx]

Additional Information

Neutrophil-mediated clinical nanodrug for treatment of residual tumor after focused ultrasound ablation

Jian Shen^1†^, Junnian Hao^1†^, Yini Chen^1^, Hairong Liu^1^, Jianrong Wu^1^, Bing Hu^1^, Yan Wang^1*^, Yuanyi Zheng^1,2*^, Xiaojun Cai^1*^

*Correspondence: [Yannanfly@126.com](mailto:Yannanfly@126.com); [zhengyuanyi@sjtu.edu.cn](mailto:zhengyuanyi@sjtu.edu.cn); [clx2j34@163.com](mailto:clx2j34@163.com);

†Jian Shen and Junnian Hao contributed equally to this work.

^1^ Shanghai Institute of Ultrasound in Medicine, Department of Ultrasound in Medicine, Shanghai Jiao Tong University Affiliated Sixth People’s Hospital, 600 Yishan Road Shanghai 200233, China

^2^ State Key Laboratory of Oncogenes and Related Genes, Shanghai Jiao Tong University School of Medicine, Shanghai, 200032, China

**Additional Methods.**

*Isolation and purification of mouse neutrophils*

Mature neutrophils were isolated from bone marrow of mice hind limbs and purified by Percoll (GE Healthcare) of gradient concentration [1]. Mice were sacrificed, and the femur and the tibia from both hind legs were removed and freed of muscle and sinew. Bone marrow was isolated by high-speed centrifugation and resuspended in phosphate-buffered saline (PBS). The obtained unicellular suspending was treated on three layer Percoll gradient solution consisting of 50%, 65%, 80% (v:v), followed by centrifugation at 800 ×g for 30 min at RT. Then, after carefully removing the cells from upper part of 80%, the neutrophils were harvest from the 65%/80% interface and the upper part of 80%. After washed by PBS three times, the mature neutrophils were prepared for future research.

The morphology of neutrophils was observed by optical microscope (Nikon) using Wright-Giemsa stain. The purity was determined using immunofluorescence double staining with fluorescence isothicyanate (FITC)-conjugated Gr-1 antibody (1 μg/ml, Biolegend) and phycoerythrin (PE)-conjugated CD11b antibody (1 μg/ml, Biolegend) by flow cytometry (Beckman). The viability of isolated neutrophils was observed by fluorescence microscope (Nikon) using Calcein-AM/PI double stain. The cell viability was calculated as N_live_/(N_live_+N_dead_) ×100%. N_live_ or N_dead_ refers to average number of five fields of view with equal size in one image.

*Cytotoxicity of PLD toward neutrophils*

The PLD we used as model drug was produced by Shanghai Fudan-Zhangjiang Biopharmaceutical Co., Ltd. The trade name of the medicinal product is LIBOD^®^. This product is a lipid system agent, the system of hydrochloric acid polyphosphate encapsulated on the surface combined with methylphenidate glycol (MPEG) liposome, which is called polyethyl glycol (PEG). It can protect the liposome from mononuclear macrophage system (MPS) recognition, thereby prolonging its circulation time in blood. This product is sterile, translucent red suspension (10 ml per bottle, the concentration of doxorubicin hydrochloride is 2 mg/ml), which is used for single dose of intravenous drip concentrate. The active ingredient of this product is doxorubicin hydrochloride, a kind of anthracycline antibiotics derived from the culture of *Streptomyces peucetius var.caesius.*

The in vitro cytotoxicity of PLD towards NEs was determined by Cell Counting Kit-8 (CCK-8, Dojindo). The isolated NEs was cultured in RPMI 1640 medium without FBS for 1 h. Then, NEs were coincubated with PLD or doxorubicin under different concentrations for 12 h. Afterwards, cells were washed gently washed by PBS thrice and adding CCK 8 solution to a final concentration of 10% (v: v) for another 1 h coculture. The absorbance was measured at a test wavelength of 450 nm by microplate reader (Thermo Fisher). The cell viability was calculated as (A_sample_-A_blank_) / (A_control_-A_blank_) ×100%.

*Stability and drug release efficiency of PLD@NEs*

The in vitro stability and drug release properties of doxorubicin from PLD@NEs were evaluated under different conditions, including the normal physiological condition, during the process of chemotaxis responding to local inflammation and at the site of inflammation. fMLP and PMA were applied to simulate the chemotactic cytokines in the blood circulation and at the site of inflammation, respectively. PLD@NEs (1 × 106 cells) were seeded in 24-well plates, and then incubated with the FBS free medium that contained fMLP (100 nM), PMA (100 nM) for different periods (0, 2, 4, 6 and 8 h). The amount of PTX released in the supernatant medium was determined by HPLC.

To further demonstrate the drug release from NEs on the PMA-induced formation of NET, PLD@NEs were treated with fMLP or PMA and observed using fluorescence microscopy (Nikon). PLD@NEs (1 × 106 cells) were seeded in confocal dishes (NEST), and then incubated with the FBS free medium containing fMLP (100 nM) or PMA (100 nM) for 0 and 8 h. Subsequently, the cells were stained with PI (5 μg/ml, Yeason) for 30 min. After washing with ice-cold PBS thrice, the cells were visualized by fluorescence microscope.

*Ultrasound monitoring*

The follow-up ultrasound was carried out using Mindray M9 Echography (Mindray Co., Shenzhen, China). The ultrasound images of tumor before and after HIFU ablation were detected using high frequency transducer with 12 MHz. The ultrasound examinations included two-dimensional ultrasound and CEUS. The US contrast agent used in the current study was SonoVue (Bracco, Milan, Italy), a suspension of stabilized sulfur hexafluoride (SF6) microbubbles in saline [2, 3]. SonoVue was suspended in 5ml saline and immediately injected to mice. The CEUS images were monitored and recorded in real time.

*In vivo biosafety evaluation*

ICR mice was used to evaluate the safety of our therapeutic strategy. Treating with different formulations as the same period as performed on hepatoma-bearing balb/nu mice, the blood samples of ICR mice were harvest. The quantities of alanine transaminase (ALT), aspartate transaminase (AST) and alkaline phosphatase in the serum were determined using corresponding assay kit (Sigma) according to the manufacturer’s instructions. Histological analyses on major organs, including heart, liver, spleen, lung and kidney, were performed by hematoxylin and eosin (H&E) stain.

**Additional references**

[1] R. Boxio, C. Bossenmeyer-Pourié, N. Steinckwich, C. Dournon, O. Nüsse, Mouse bone marrow contains large numbers of functionally competent neutrophils. Journal of leukocyte biology 75, 604-611 (2004).

[2] T. Albrecht, J. Hohmann, A. Oldenburg, J. Skrok, K. J. Wolf, Detection and characterisation of liver metastases. European radiology 14 Suppl 8, P25-33 (2004).

[3] E. Leen, W. J. Angerson, S. Yarmenitis, G. Bongartz, M. Blomley, A. Del Maschio, V. Summaria, G. Maresca, C. Pezzoli, J. B. Llull, Multi-centre clinical study evaluating the efficacy of SonoVue (BR1), a new ultrasound contrast agent in Doppler investigation of focal hepatic lesions. European journal of radiology 41, 200-206 (2002).

**Additional figures**


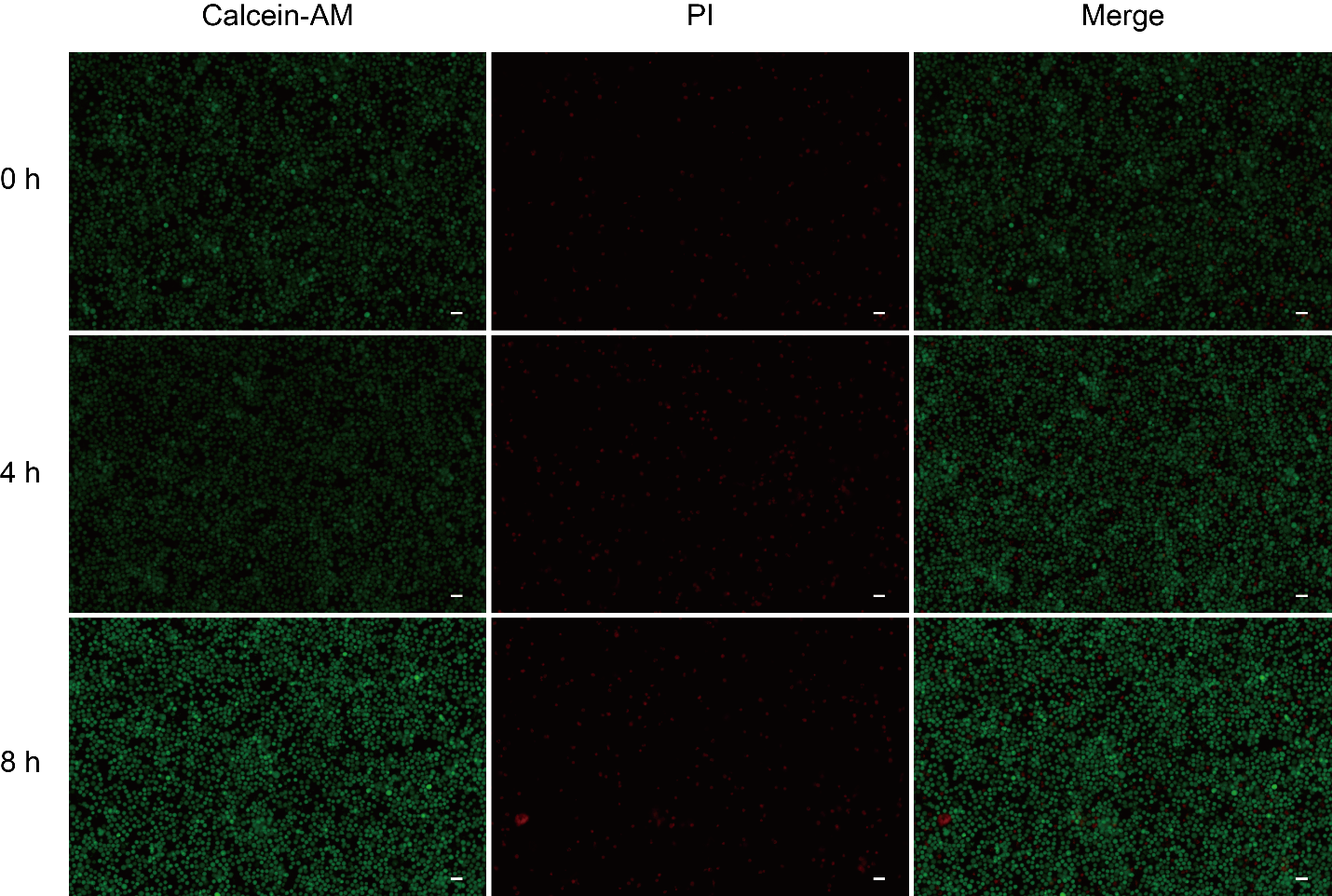


Figure S1. Cell viability monitoring of neutrophils after loading with PLD for 0, 4 or 8 h. Scale bar: 10 μm.


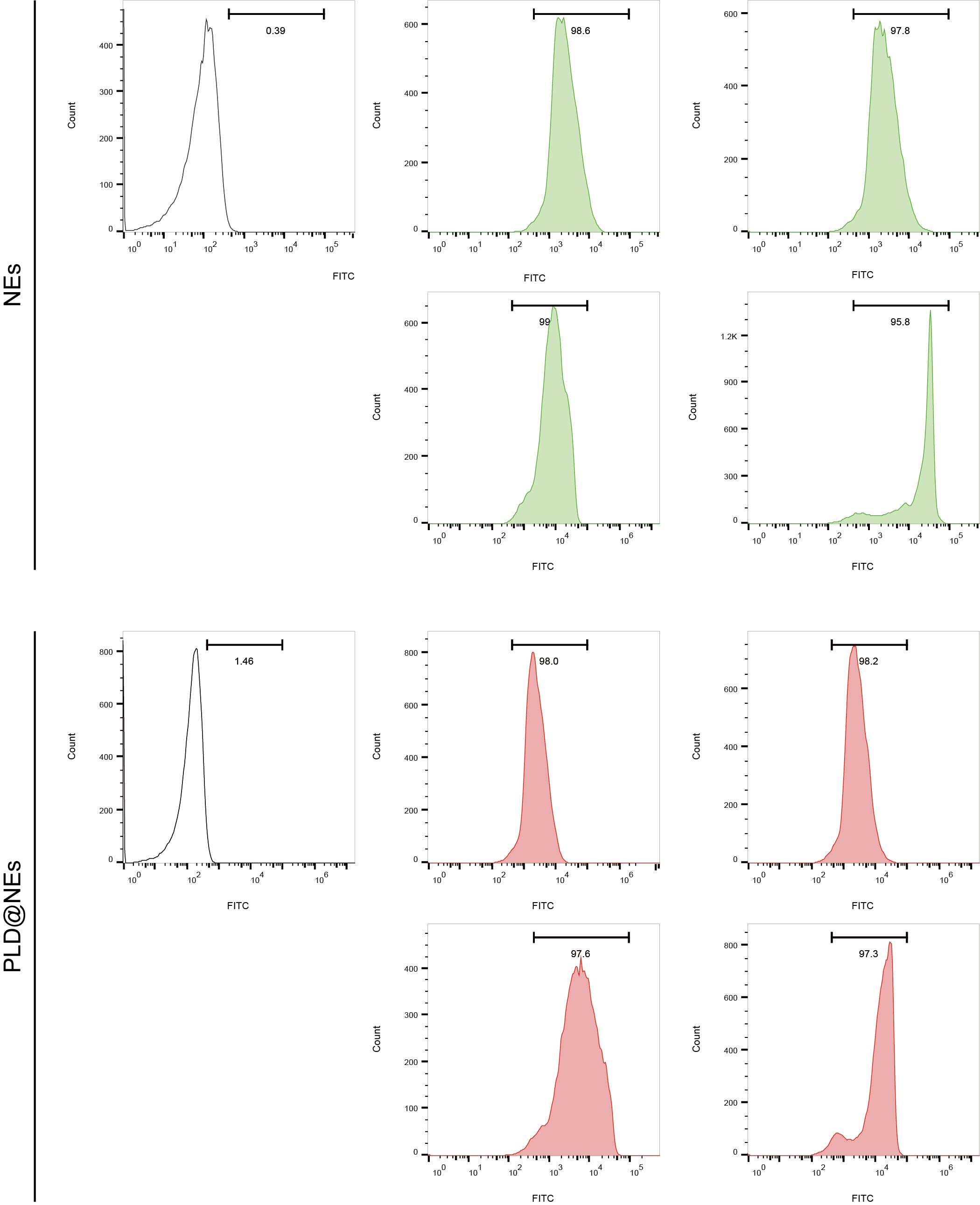
Figure S2. The CD11b expression level of PLD@NEs and NEs in the presence of fMLP (100 nM). The fluorescence intensity of neutrophils stained by the FITC conjugated CD11b antibody indicated the expression level of CD11b on cell membrane.

**
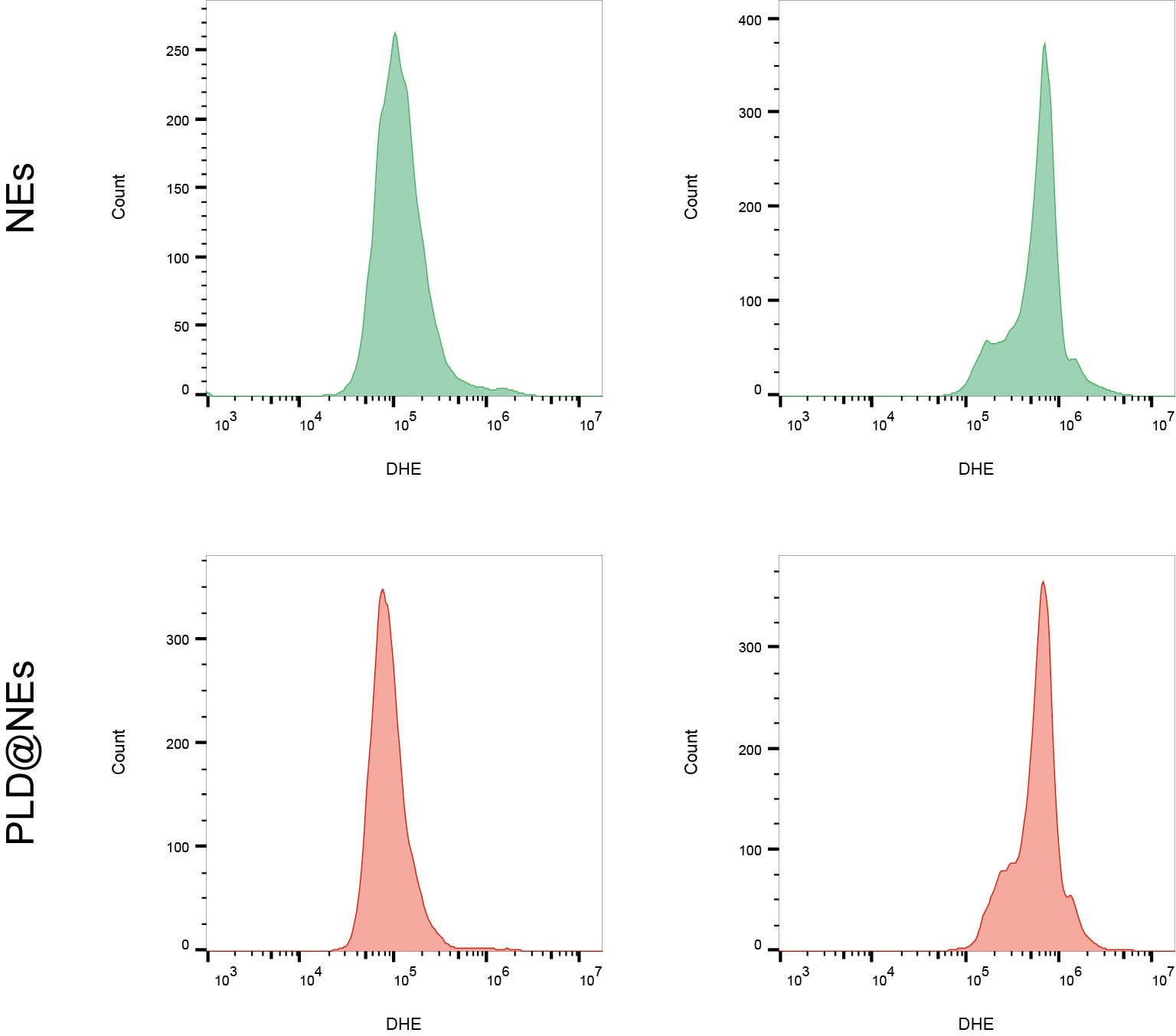
Figure S3. The superoxide generation of PLD@NEs and NEs in the presence of fMLP (100 nM).** The fluorescence intensity of neutrophils stained by DHE indicated the level of superoxide.

**
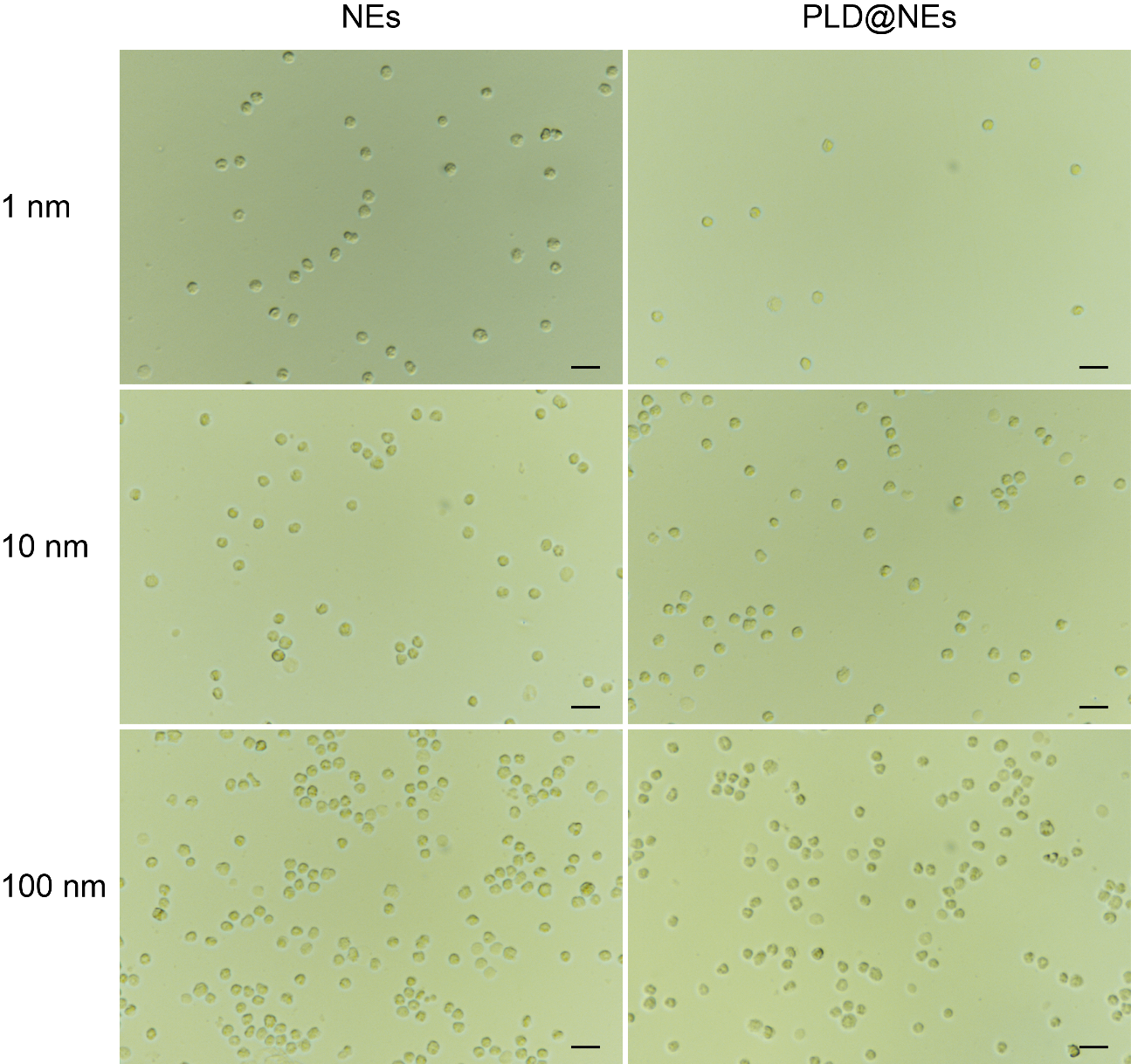
Figure S4. Bright fields (BF) observation of neutrophils transmigrated from upper chamber to lower chamber in the transwell plate under the stimulating of fMLP.** Scale bar: 20 μm.

**F
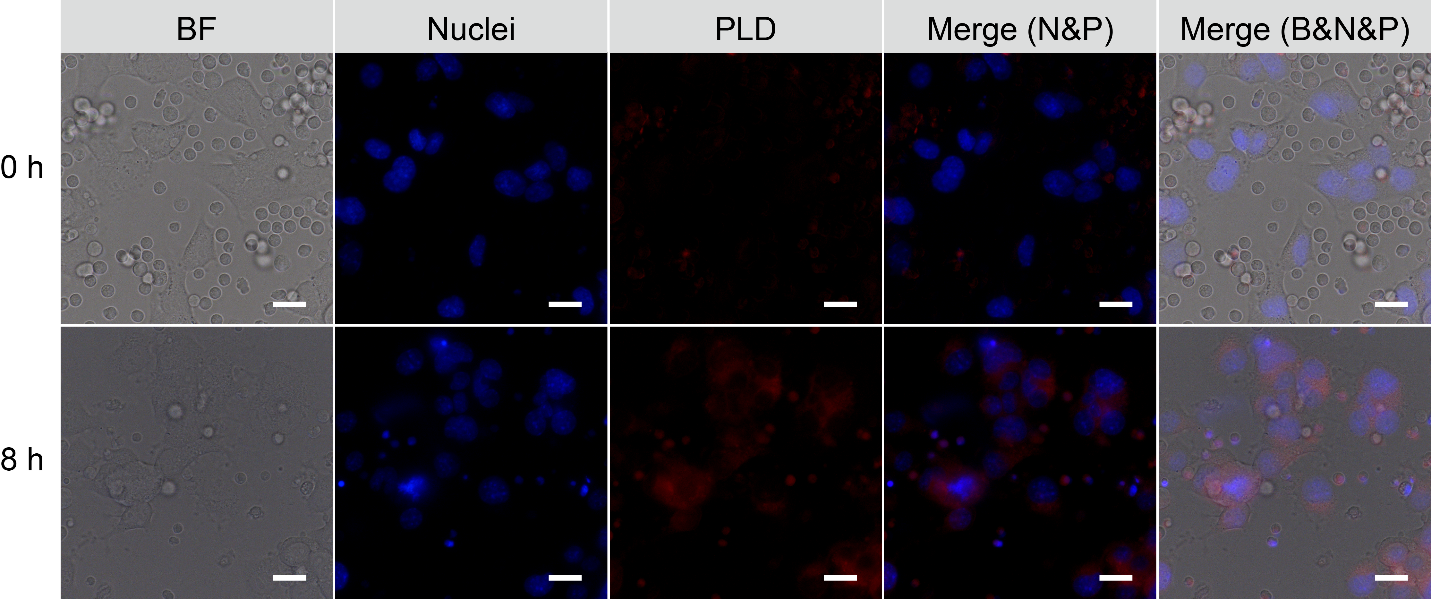
igure S5. Fluorescence images of Hepa 1-6 cells after incubation with PLD@NEs treated with PMA (100 nM) for 8 h.** The nuclei of Hepa 1-6 cells were stained with Hoechst 33342, and the released DNA segments were stained with PI. Scale bar: 20 μm.


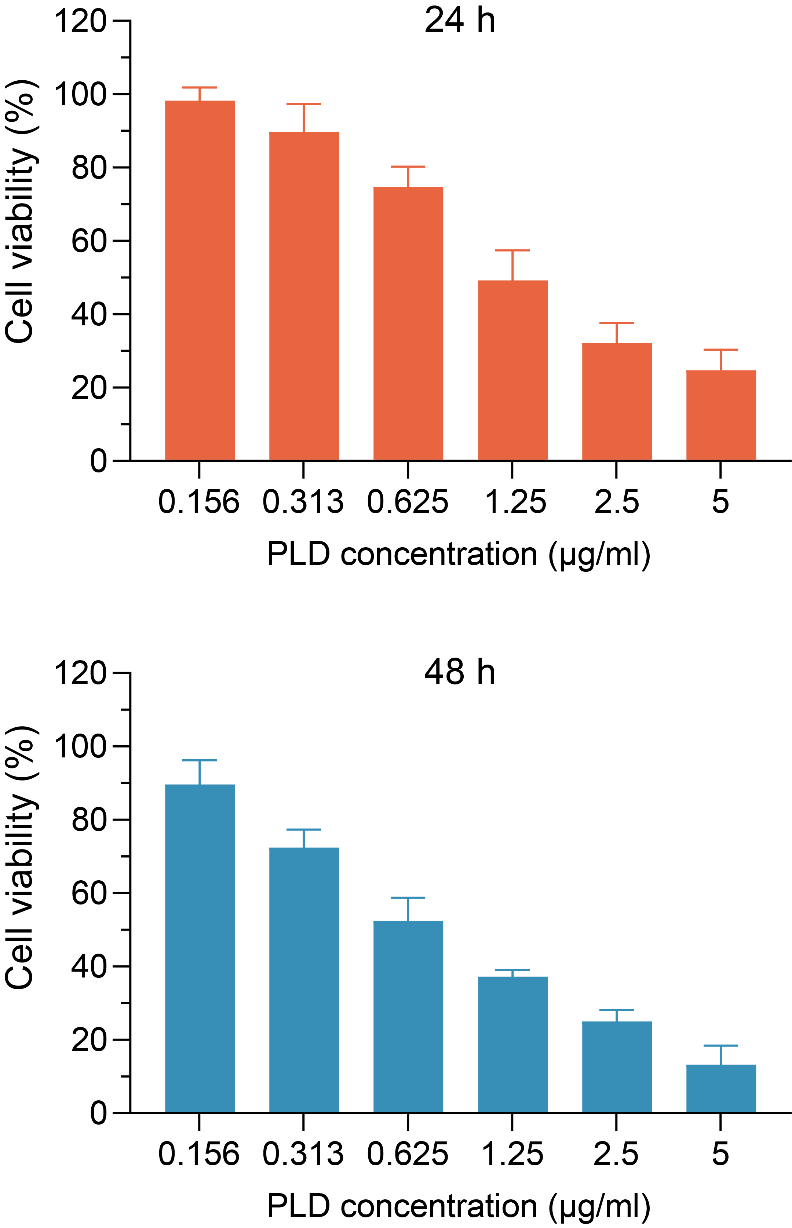
Figure S6. Cytotoxicity of PLD@NEs against 4T1 cells. The cell viability was detected by CCK8 assay after incubating supernatant collecting from PMA treated PLD@NEs with 4T1 for 24h/48h. Data are shown as mean ± SD. n=5; *P＜0.05, **P＜0.01, ***P＜0.001.


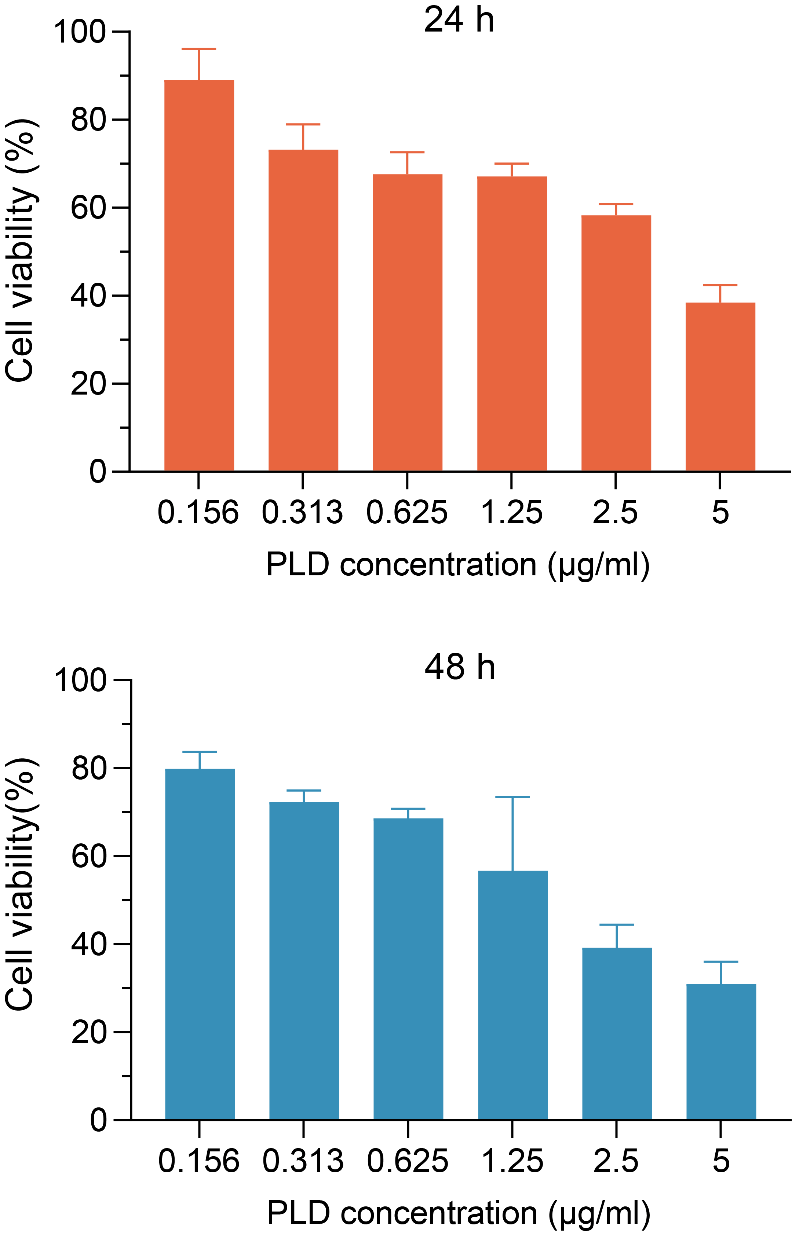
Figure S7. Cytotoxicity of PLD@NEs against HepG2 cells. The cell viability was detected by CCK8 assay after incubating supernatant collecting from PMA treated PLD@NEs with HepG2 for 24 h/48 h Data are shown as mean ± SD. n=5; *P＜0.05, **P＜0.01, ***P＜0.001.


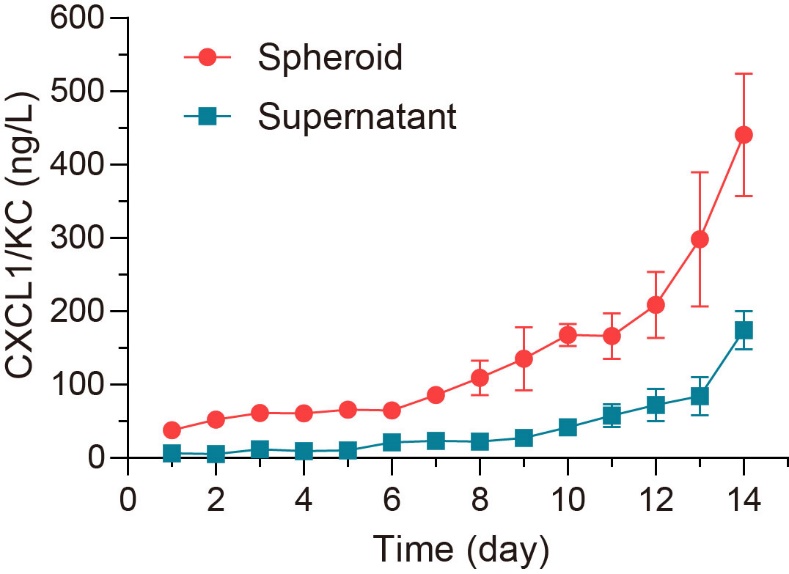
Figure S8. Expression of CXCK1/KC in the medium (blue) and in the hepa 1-6 multicellular spheroid (red) during 14 days of culture. Data are shown as mean ± s.d. n=3.

**
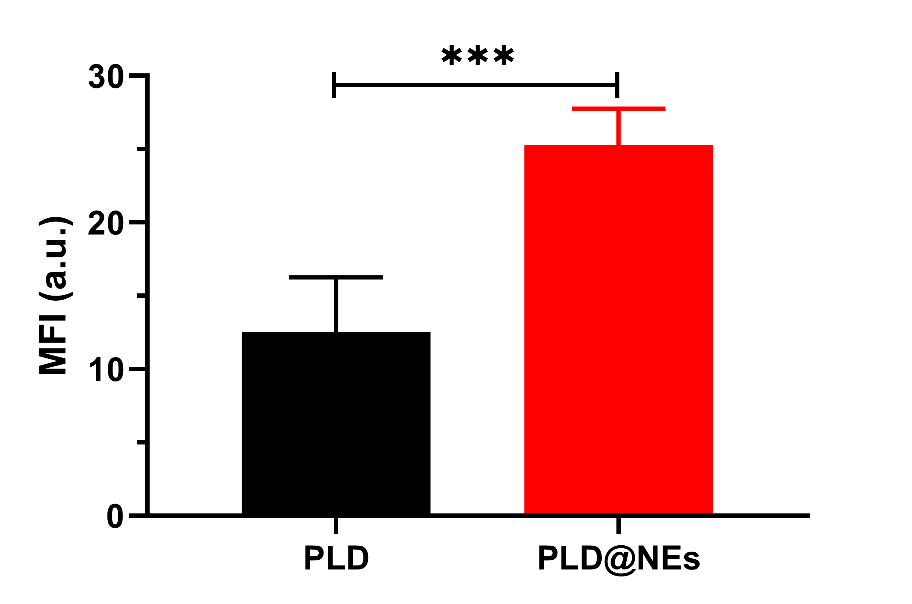
Figure S9. Semi-quantitative analysis of tumor permeability after incubating 3D hepa1-6 tumor spheroids with PLD or PLD@NEs for 8 h.**
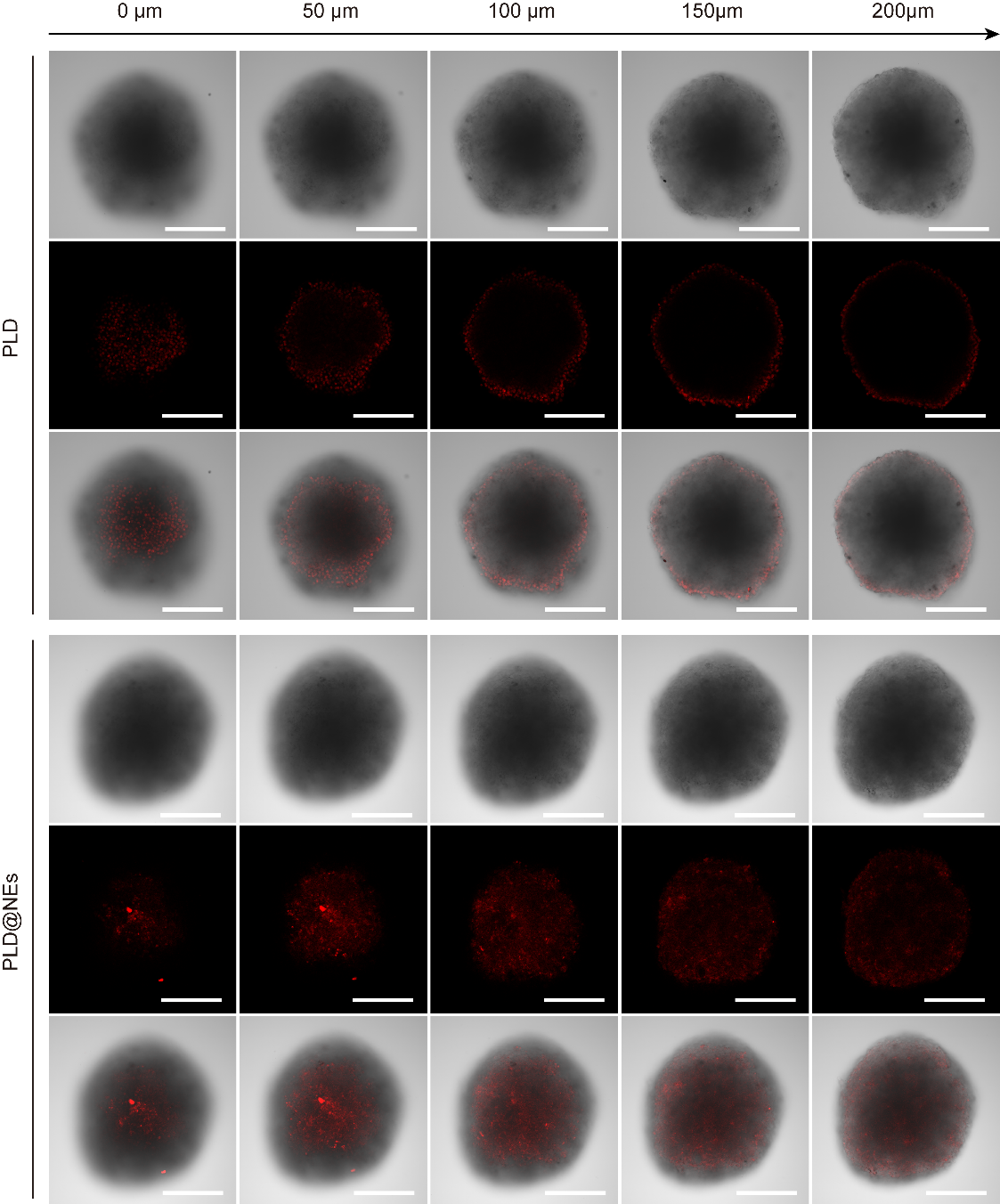
**Figure S10. Tumor permeability of PLD@NEs after incubation with 3D hepa1-6 tumor spheroids for 8h.** CLSM images were obtained from the surface to the middle of tumor spheroids in a Z-stack thickness of 50 μm. Scale bar: 200 μm.


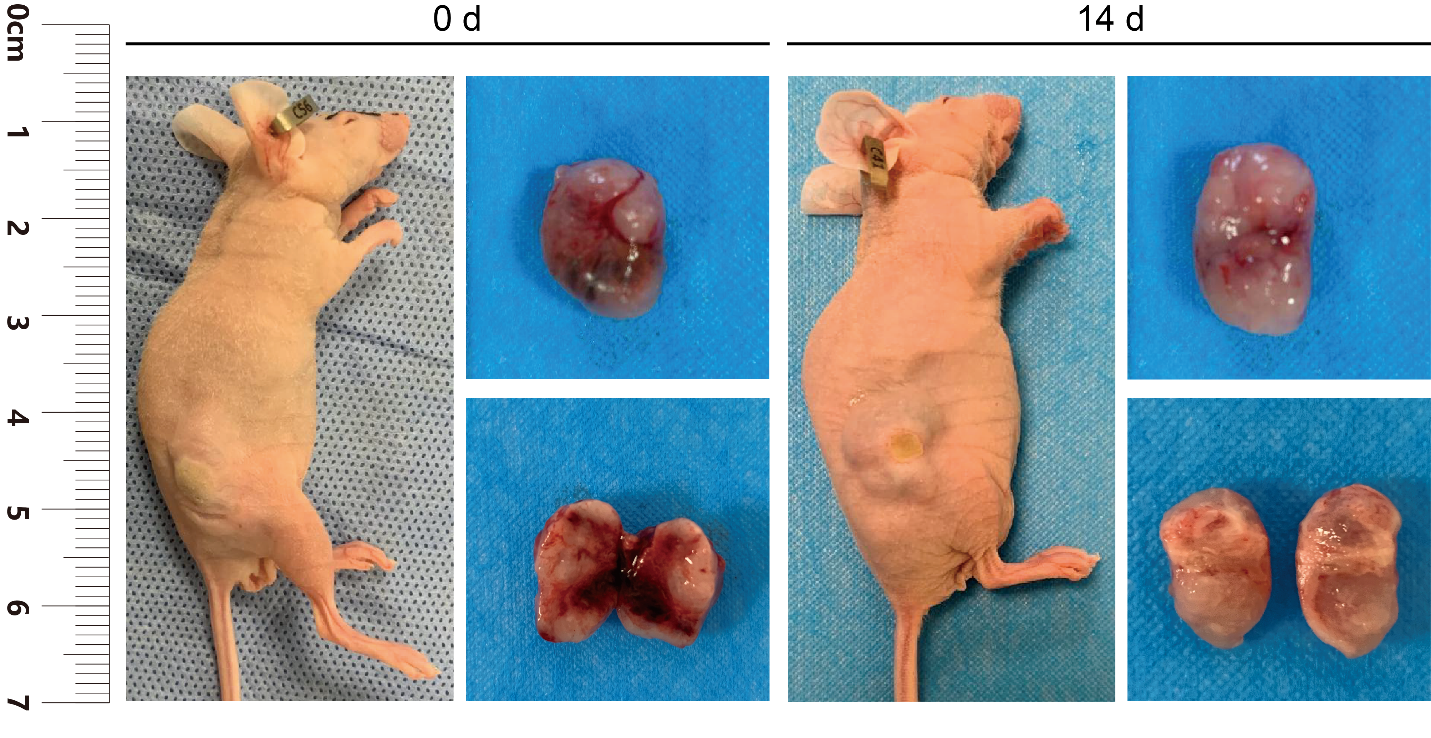
Figure S11. In vivo and ex vivo tumor tissues after HIFU ablation. On day 0, the HIFU lesion was observed as pale area surrounded by hyperemia. On day 14, the HIFU lesion was observed as pale area without hyperemia and there is sharp differentiation between ablated tissue and normal tumor tissue. Mild skin injuries were observed of mice after HIFU treatment using our therapeutic mold for mice.

**
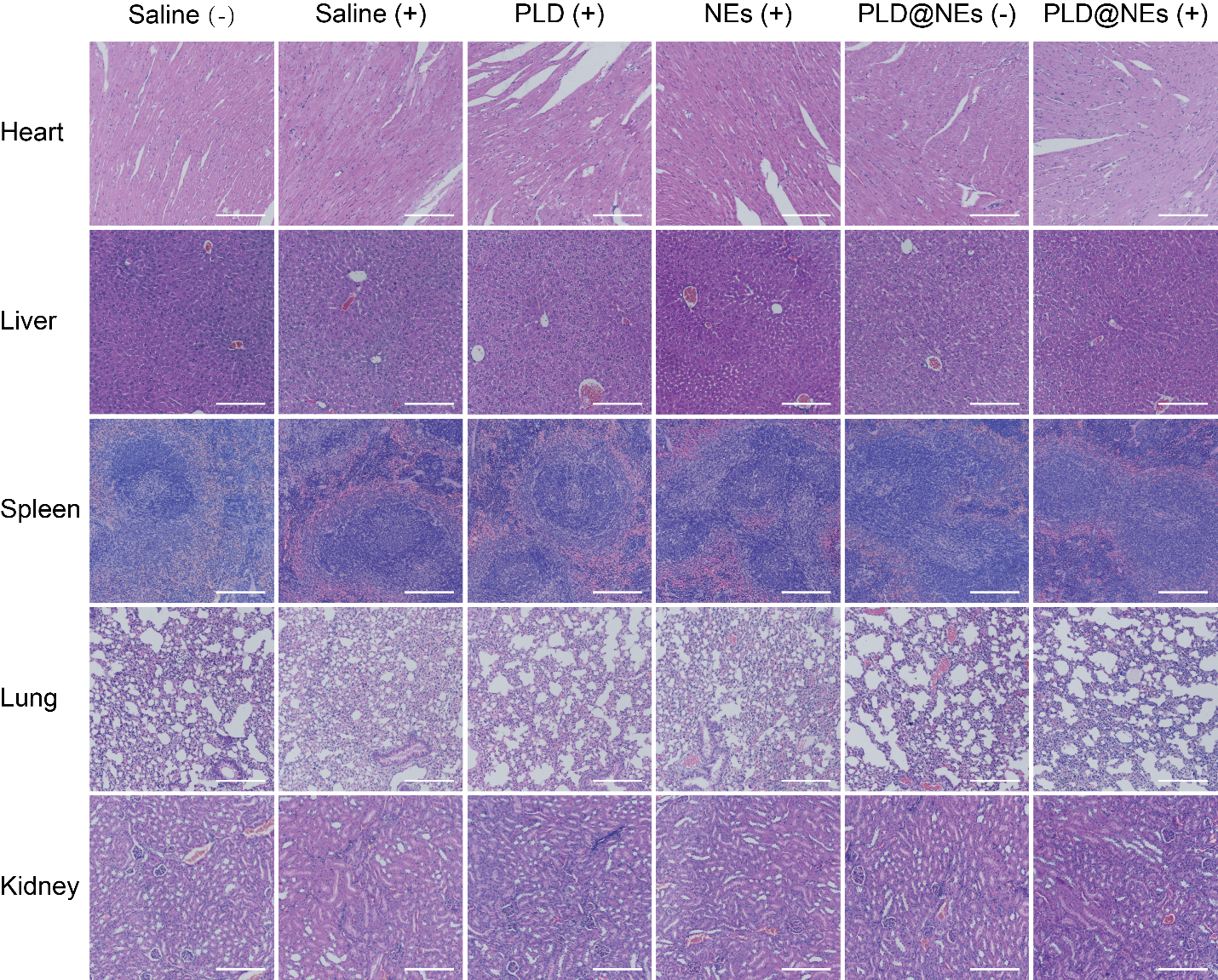
Figure S12. Histology (H&E) of major organs from hepatoma-bearing mice after combined treatment of HIFU and different formulations.** No noticeable pathological abnormality of major organs was observed of PLD@NEs treated group. Scale bar: 200μm.


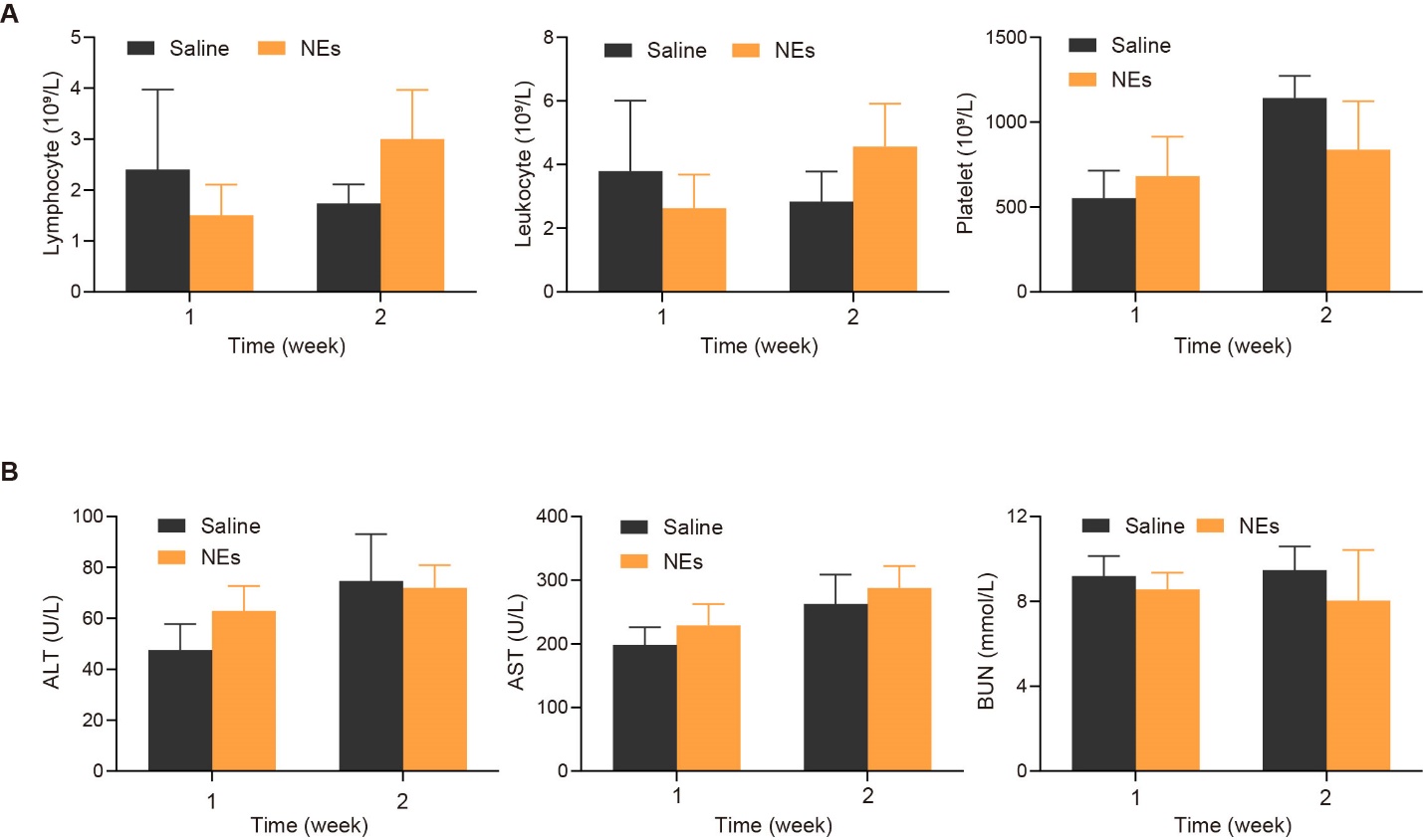
Figure S13. Biosafety evaluation of PLD@NEs injected to the Hepa 1-6 tumor-bearing after HIFU ablation. (A) Haemocytes counts including lymphocytes, leukocytes and platelets at 1st week and 2nd week of mice. n=3. (B) Blood biochemical indexes including ALT, AST and BUN at 1st week and 2nd week of mice. n=3. ALT: alanine aminotransferase. AST: aspartate aminotransferase. BUN: blood urea nitrogen.

**Movie S1.** **Dynamic contrast enhanced ultrasound (CEUS) images of tumor after HIFU.**
